# Assessment of Fecal Microbiota in Healthy Dogs and Dogs with Cutaneous Mast Cell Tumors Treated with Electrochemotherapy Combined with Gene Electrotransfer of IL-12

**DOI:** 10.3390/vetsci13030241

**Published:** 2026-03-01

**Authors:** Anja Lisjak, Bruna Correa Lopes, Rachel Pilla, Ana Nemec, Urša Lampreht Tratar, Jan S. Suchodolski, Nataša Tozon

**Affiliations:** 1 Small Animal Clinic, Veterinary Faculty, University of Ljubljana, 1000 Ljubljana, Slovenia; ana.nemec@vf.uni-lj.si (A.N.); ulampreht@onko-i.si (U.L.T.); natasa.tozon@vf.uni-lj.si (N.T.); 2 Gastrointestinal Laboratory, Department of Small Animal Clinical Sciences, College of Veterinary Medicine & Biomedical Sciences, Texas A&M University, College Station, TX 77843, USA; brunalopes@tamu.edu (B.C.L.); rpilla@exchange.tamu.edu (R.P.); jsuchodolski@cvm.tamu.edu (J.S.S.); 3 Department of Veterinary Pathology, Hygiene and Public Health, University of Milan, 20122 Milan, Italy; 4 Department of Experimental Oncology, Institute of Oncology Ljubljana, 1000 Ljubljana, Slovenia

**Keywords:** MCT, canine microbiome, electrochemotherapy, gene electrotransfer, dysbiosis index, shallow shotgun sequencing, metagenomics

## Abstract

Cancer is a major health concern, with its incidence rate continuing to increase. There is growing interest in the microbiota and its role in carcinogenesis, as it significantly influences physiological and pathological processes. Various aspects of the microbiome have been shown to have both anti-tumor and pro-tumor effects. Advances in techniques such as high-throughput DNA sequencing have greatly improved our understanding of microbial populations in the human and canine gut. We aimed to (1) characterize the intestinal microbiota of healthy dogs and dogs with cutaneous mast cell tumors (MCTs), (2) assess changes in the intestinal microbiota of dogs undergoing electrochemotherapy (ECT) combined with gene electrotransfer (GET) of the IL-12 plasmid (IL-12), and (3) explore possible associations with the expression of immune markers Programmed cell death protein 1 (PD-1), Programmed death-ligand 1 (PD-L1), and Granzyme B (GZMB) in MCT tissue. Stool samples were collected from healthy dogs (*n* = 24) and dogs with MCTs (*n* = 24) before and after ECT and IL-12 GET. DNA was extracted from the samples, and shallow shotgun sequencing was performed. Immunohistochemistry was performed on the tumors to assess the expression of PD-1, PD-L1, and GZMB. The dysbiosis index, alpha diversity, and beta diversity did not differ between groups. Regarding microbial composition, *Bifidobacterium animalis*, *Corynebacterium variabile*, *Lactobacillus johnsonii*, *Pediococcus pentosaceus*, *Streptococcus anginosus*, *Streptococcus equinus*, *Streptococcus intermedius*, *Clostridium thermobutyricum*, *Megasphaera elsdenii*, and *Anaerobiospirillum* sp. were found in lower relative abundance in feces of dogs with MCTs, while *Bacteroides togonis*, *Lactobacillus amylolyticus*, *Prevotella* sp. *CAG:279*, and *Megamonas hypermegale* were more abundant compared to healthy dogs. Our study provides further insight into the composition of the gut microbiota in dogs with MCTs, where ECT and IL-12 GET did not lead to major shifts. We were unable to establish any association between the expression of immune markers and the microbiota.

## Simple Summary

Cancer remains a major health concern, and increasing attention is being given to the role of the microbiota in cancer development and progression. The gut microbiome can influence both normal physiological processes and disease, including tumor growth. In dogs, cutaneous mast cell tumors are among the most common skin cancers, yet the relationship between this disease and the intestinal microbiota is still not well understood. In this study, we examined the gut microbiota of healthy dogs and dogs diagnosed with mast cell tumors. We also investigated whether electrochemotherapy combined with interleukin-12 gene electrotransfer affected the microbiome. Additionally, we evaluated whether differences in gut bacteria were related to the expression of key immune markers in tumor tissue. Overall microbial diversity and the dysbiosis index did not differ significantly between groups. Dogs with mast cell tumors had lower levels of certain beneficial bacteria and higher levels of others compared to healthy dogs. However, treatment did not lead to major shifts in the microbiome. We also found no clear links between gut microbiota composition and tumor immune marker expression. These results provide new insight into the gut microbiota of dogs with mast cell tumors and suggest that both the disease and its treatment cause only subtle microbial changes.

## 1. Introduction

The complexity of the metagenome and the diversity of the microbiota in health and disease have generated interest in human and veterinary medicine in understanding the precise role of intestinal dysbiosis in cancer [1]. The canine model of spontaneous and non-spontaneous cancer offers a unique opportunity to investigate the influence of the intestinal microbiome on cancer development, treatment, and prognosis [2]. The gut microbiome has been shown to influence the immune response against cancer [3]. The presence of certain bacteria plays a crucial role in the development of chronic inflammation, production of genotoxic metabolites, disruption of cell-cycle regulation, and modulation of host immune responses, all of which can be involved in carcinogenic processes [4,5,6,7,8].

Mast cell tumors (MCTs) are among the most common skin tumors in dogs, accounting for 11% of skin cancer cases in this species [9]. The biological behavior of MCTs in dogs is highly variable. According to Patnaik et al., well-differentiated MCTs, commonly referred to as low-grade, tend to have a milder clinical course, while less-differentiated tumors, referred to as high-grade, are more aggressive [9,10]. Metastases primarily affect locoregional lymph nodes and may later spread to the spleen, liver, and other organs. In general, dogs with mast cell tumors, regardless of grade, that exhibit regional lymph node involvement have a poorer prognosis [9].

One treatment method for MCT in dogs is electrochemotherapy (ECT). ECT is a local therapy that combines chemotherapy with the application of specific electrical pulses, allowing antineoplastic drugs to penetrate cells [11]. Currently, cisplatin and bleomycin are used clinically to treat MCTs [12]. ECT increases the local absorption of antineoplastic drugs, which remain inside the cell after membrane permeability is restored, maximizing their cytotoxicity. An advantage of ECT is that its effects are limited to tissues exposed to the electrical pulses, reducing the risk of chemotherapeutic side effects [13,14,15,16,17].

ECT can be combined with gene electrotransfer therapy (GET), in which plasmid DNA encoding a therapeutic gene (transgene) is delivered into cells via electroporation, a method similar to ECT that enables transgene expression. One of the transgenes used by GET in veterinary oncology [18,19] and human oncology [20,21] is interleukin 12 (IL-12). IL-12 is a proinflammatory cytokine that promotes the production of interferon gamma, which plays a crucial role in the antitumor immune response by stimulating antigen-presenting cells and cytotoxic cells, such as T lymphocytes and natural killer cells [22]. Intratumoral administration of IL-12 GET increases IL-12 production, activating a specific immune response against tumor antigens [17].

A key feature of carcinogenesis is immune escape, in which cancer cells evade recognition by the immune system. One mechanism is the overexpression of immune checkpoint ligands, such as PD-1 and PD-L1, which leads to T cell anergy. Cancer immunotherapies targeting these receptors offer a promising approach to treating cancers in both human and veterinary medicine [23]. PD-L1 is expressed in many types of tumor tissues in dogs, including mast cell tumors [24,25,26]. Expression of PD-1 by tumor-infiltrating lymphocytes has been observed in canine melanoma and lymphoma [24,26,27,28]. The significance of PD-1 and PD-L1 expression in low-grade MCTs remains unclear.

Alterations in fecal microbiota composition have been observed in dogs with multicentric [29,30] and intestinal lymphoma [31], lymphoid and nonlymphoid tumors [32], colorectal epithelial tumors [33], and mammary tumors [34]. Few studies have examined changes after therapy, focusing mainly on chemotherapy [1]. A recent study by Aragon et al. [35] demonstrated that chemotherapy with vincristine and prednisolone worsens gut dysbiosis and alters microbial function in dogs with lymphoma. The study reported a significant increase in the dysbiosis index (DI) after chemotherapy, along with a marked decrease in fecal *Peptacetobacter hiranonis*. Additionally, 16S rRNA gene sequencing revealed a significant reduction in *Enterococcaceae* abundance after chemotherapy [35].

Recent studies have identified the human gut microbiota as a potential predictor of response to immune checkpoint inhibitors (ICIs) [36]. Several studies have linked the presence of *Faecalibacterium prausnitzii* in the gut microbiota to improved responses to ICIs in human melanoma patients. Higher abundances of this bacterium are associated with longer progression-free survival and enhanced antitumor immune responses [37]. In human patients with non-small cell lung cancer, the presence of *Akkermansia muciniphila* has been correlated with better clinical outcomes following anti-PD-1 therapy. This bacterium appears to enhance the efficacy of ICIs by modulating the host immune response [38]. The Ruminococcaceae family has also been associated with stronger antitumor immune responses in patients treated with PD-1/PD-L1 inhibitors. Higher abundances of these bacteria are linked to improved treatment outcomes [39].

Therefore, this study aimed to (1) characterize the intestinal microbiota of dogs with cutaneous MCTs compared to healthy control dogs, (2) assess changes in the intestinal microbiota of dogs undergoing ECT and IL-12 GET, and (3) examine the expression of the immune markers PD-1, PD-L1, and GZMB in MCT tissue.

## 2. Materials and Methods

### 2.1. Study Population, Sample Collection and Inclusion Criteria

The study included 48 client-owned dogs. The healthy control group consisted of 24 clinically healthy dogs, aged 24 to 172 months and weighing 3 to 42 kg, including 10 intact males, 1 neutered male, 5 intact females, and 8 neutered females. The MCT group included 24 dogs aged 40 to 142 months and weighing 7 to 48.5 kg: 3 intact males, 7 neutered males, 4 intact females, and 10 neutered females. The age, weight, sex, and breeds of dogs in both groups are shown in Table 1. Stool samples were collected from all 48 dogs.

Differences in age, weight, and sex between groups were not statistically significant (Table 2).

Dogs with MCTs underwent ECT and IL-12 GET using either bleomycin or cisplatin, administered intratumorally or intravenously as chemotherapeutic agents, along with the plasmids pORFcaIL12ORT or pCMVcaIL12, as previously reported [18,40]. All samples were collected at the Small Animal Clinic, Veterinary Faculty, University of Ljubljana, Slovenia.

The following inclusion criteria applied to all dogs: patients must not have received antibiotics, proton pump inhibitors, or corticosteroids for at least two months before fecal sample collection. All owners were required to complete a questionnaire about dogs’ health, activity, medication, and diet (Supplementary Table S1), and to sign a written consent form. Full physical examinations and blood tests were performed before sampling. The basic blood examination included a complete blood count with differential white blood cell counts, performed using an automated hematology analyzer (Advia 120, Siemens, Munich, Germany). An automated chemistry analyzer, RX-Daytona (Randox, Crumlin, UK), was used to determine the following biochemical parameters: urea, creatinine, alanine aminotransferase and alkaline phosphatase.

The owners of animals with MCTs voluntarily participated in the clinical trial and chose ECT and IL-12 GET, declining other standard treatments (such as surgery and/or radiotherapy) at enrollment due to financial reasons, unavailability of other treatment options, invasiveness (such as limb amputation or extensive reconstructive surgery), or other reasons.

Before treatment, the clinical stage of MCT patients was determined by abdominal ultrasonography [41,42], as well as fine-needle aspiration biopsy and cytological examination of the spleen and liver to further exclude metastatic disease.

### 2.2. ECT and IL-12 GET Treatment Protocol

ECT and IL-12 GET procedures in dogs with MCTs were performed under general anesthesia. Dogs received intravenous premedication with midazolam (Midazolam Torrex, Torrex Pharma GesmbH, Vienna, Austria; 0.1 mg/kg) and butorphanol (Butomidor, Richter Pharma AG, Wels, Austria; 0.3 mg/kg). Anesthesia was induced with propofol (Propomitor, Animalcare, York, UK; 3–6 mg/kg) and maintained with sevoflurane (Sevorane, AbbVie Inc., North Chicago, IL, USA) in 100% oxygen. During the procedure, Hartmann’s solution (B. Braun Melsungen AG, Melsungen, Germany) was administered at 5 mL/kg/h. After induction, clemastine (Tavegyl, GlaxoSmithKline Dungarvan Ltd., Co. Waterford, Ireland; 0.05 mg/kg) was injected intramuscularly.

To minimize the risk of mast cell degranulation and potential histamine-related reactions, the hair around the nodules was carefully trimmed without unnecessary manipulation of the tumor. Tumor volume was calculated using three perpendicular diameter measurements taken with a caliper, applying the formula V = a × b × c × π/6. This measurement guided the appropriate dosing of the cytostatic drug for intratumoral administration. The largest tumor diameter was used to assess treatment efficacy according to the response evaluation criteria for solid tumors in dogs [12,18,43].

All patients followed the same electrochemotherapy (ECT) protocol. ECT was performed using either direct intratumoral administration of cisplatin (Cisplatin Accord, Accord Health Care, London, UK) or intravenous bleomycin (Bleomycin Medac, Medac GmbH, Hamburg, Germany). Cisplatin (1 mg/mL) was injected intratumorally at a dose of 1 mg/cm^3^ for tumors smaller than 1 cm^3^ and 0.5 mg/cm^3^ for larger tumors. Bleomycin was prepared at 3 mg/mL and administered intravenously at 0.3 mg/kg. Cisplatin was the first-choice drug unless the patient had multiple or bleeding tumors [12,18]. GET of IL-12 was performed as previously described [18,40]. Regardless of the ECT modality, plasmid DNA was injected 5 min before the EP pulses. Plasmid DNA (pCMVcaIL-12 or pORFcaIL-12-ORT) was injected intratumorally at a dose of 2 mg per patient.

After four weeks, treatment responses were assessed and categorized according to the Response Evaluation Criteria in Solid Tumors (RECIST) as follows: complete response (CR)—disappearance of the nodule; partial response (PR)—at least a 30% decrease in the sum of the diameters of the target lesion, using the baseline sum diameters as reference; progression of disease (PD)—at least a 20% increase in the sum of the diameters of the target lesions, using the smallest sum recorded as reference [44].

The clinical trial was approved by the Ethics Committee of the Ministry of Agriculture, Forestry, and Food of the Republic of Slovenia (approval no. U34401-19/2021/8, date of issuing: 18 October 2021) and the Scientific Committee for the Deliberate Release of GMOs of the Ministry of Environment and Spatial Planning (approval no. 35419-1/2021-2550-8, date of issuing: 20 July 2021).

### 2.3. Stool Sample Collection

Stool samples were collected directly from the rectum using a gloved finger before treatment, immediately after anesthesia induction, and again one month after treatment in awake animals. For healthy control dogs, stool samples were also obtained from awake patients using a gloved finger. All samples were collected directly from the rectum, placed in sterile sampling tubes, and immediately stored at −80 °C for further analysis at the Texas A&M Gastrointestinal Laboratory.

### 2.4. DNA Extraction and Dysbiosis Index

Total fecal DNA was extracted using the QIAamp PowerFecal Pro DNA Kit (QIAGEN, Hilden, Germany) and an automated extraction system (Thermo KingFisher Flex Magnetic Particle Purification 96 PCR Isolation System, Thermo Fisher Scientific, Waltham, MA, USA), following the manufacturer’s instructions.

The canine dysbiosis index (DI) is a qPCR-based method that measures the abundance of seven bacterial taxa and the total number of bacteria. These bacterial abundances are combined using a mathematical algorithm, known as the DI. Abundances of *P. hiranonis*, *Faecalibacterium*, *E. coli*, *Fusobacterium*, *Turicibacter*, *Streptococcus*, *Blautia*, and total bacteria were assessed for the DI. In addition, the abundances of *Bifidobacterium* and *Bacteroides* were also measured. The qPCR assays were performed as described elsewhere [45].

### 2.5. Boostershot^®^ Shallow Shotgun Metagenomic Sequencing

Microbial DNA from fecal samples was extracted using the MoBio PowerSoil^®^ DNA Isolation Kit (MoBio Laboratories, Carlsbad, CA, USA) and quantified with the Quant-iT PicoGreen dsDNA Assay Kit (Thermo Fisher Scientific Inc., Waltham, MA, USA). For sequencing library preparation, the Nextera XT DNA Library Preparation Kit (Illumina Inc., San Diego, CA, USA) was used prior to pooling the libraries. After pooling, SPRI bead purification and concentration were performed using SpeedBeads Magnetic Carboxylate Modified Particles (Cytiva Life Sciences, Marlborough, MA, USA). The pooled libraries were then denatured with NaOH, diluted, and spiked with 2% PhiX.

Metagenomic sequencing was performed on an Illumina NovaSeq 6000 System (Illumina, Inc., San Diego, CA, USA) using single-end 1 × 100 base pair read chemistry. Samples were multiplexed on the sequencer, converted to FASTQ files, and filtered for low quality (Q-score < 30) and length (<50). Adapter sequences were further trimmed, and all sequences were trimmed to a maximum length of 100 bp before alignment. Raw sequences were deposited in the NCBI Sequence Read Archive before analysis with established pipelines. For taxonomic classification, FASTA sequences were aligned to a curated database containing all representative genomes in the NCBI RefSeq, a representative genome collection for prokaryotes, with additional manually curated bacterial strains [46].

Alignments were performed at 97% identity and compared to reference genomes. Each input sequence was compared to every reference sequence in the Diversigen DivDB-Dog database using fully gapped alignment with BURST. Ties were resolved by minimizing the total number of unique Operational Taxonomic Units (OTUs). Input sequences were assigned to the lowest common ancestor for taxonomy assignment that was compatible with at least 80% of the reference sequences. OTUs representing fewer than one million species-level markers, OTUs with less than 0.01% of their unique genome regions matching, and OTUs with less than 0.1% of the whole genome matching were discarded.

For downstream analysis, normalized and filtered tables were used in QIIME2 [47]. Alpha diversity was assessed by the number of species, Shannon–Wiener index, Chao1 index, and observed OTUs using a rarefied OTU table. Beta diversity was evaluated using principal coordinate analysis (PCoA) plots based on Bray–Curtis dissimilarity.

### 2.6. Immunohistochemistry for Detection of PD-1 and PD-L1

Tumor samples were obtained by excisional or incisional biopsy and fixed in 10% formaldehyde and then embedded in paraffin at the Institute of Pathology, Veterinary faculty in Ljubljana. The samples were then transported to the Ljubljana Institute of Oncology, where they were sectioned into 2 μm-thick slices using a sliding microtome (Leica RM2235, Leica, Wetzlar, Germany). For immunohistochemical staining, appropriate polyclonal antibodies against human PD-1 (Anti PD-1 antibody PA5-32543, Thermo Fisher Scientific, Waltham, MA, USA; 1:250), human PD-L1 (Anti PD-L1 antibody ab233482, Abcam, Cambridge, UK; 1:100) and monoclonal antibody against human Granzyme B (Anti GZMB antibody ab4059, Abcam, Cambridge, UK, 1:1000) were used, as previously reported [18]. For all samples, one slice was left unstained as a negative control, and canine lymph nodes were used as a positive control. To visualize the staining, a commercially available rabbit-specific HRP/DAB detection IHC kit (ab64261, Abcam) was used according to the manufacturer’s instructions. Briefly, after the slides were deparaffinized and hydrated, antigen retrieval was performed using 10 mM sodium citrate buffer (Sigma-Aldrich, St. Louis, MO, USA) with 0.05% Tween^®^ 20 detergent (VWR, West Chester, PA, USA, CSP) in a microwave oven for 25 min. The slides were then incubated overnight at 4 °C with rabbit antibodies against PD-1, PD-L1 and GZMB. Positive staining was visualized using DAB chromogen in combination with streptavidin peroxidase. Hematoxylin was used as a nuclear counterstain.

The slides were evaluated using a light microscope (BX-51, Olympus, Hamburg, Germany). At least five photographs of the tumor were taken from each slide with a camera connected to the microscope (DP72 CCD camera, Olympus) at 400x magnification (40x objective, numerical aperture 0.85). Three independent reviewers, who had no prior knowledge of the included patients, assessed the images.

The method recommended by Sampedro-Núñez and colleagues was used [48]. Briefly, for each image of each slide, the percentage of stained cells was scored from 0 to 4 (<5% stained cells, 1 point; 6–25% stained cells, 2 points; 26–50% stained cells, 3 points; >50% stained cells, 4 points), and the intensity of staining was scored from 0 to 3 (negative staining, 0 points; mild staining, 1 point; moderate staining, 2 points; strong staining, 3 points). The points for percentage and intensity were summed, and the average of all scorers was calculated to determine the final score for PD-1 and PD-L1 expression. Granzyme B (GZMB) was analyzed by counting the number of GZMB-positive cells in the captured images. The results were expressed as GZMB-positive cells per 100 cells [18].

### 2.7. Statistical Analysis

Descriptive statistics summarized the characteristics of the study population. Continuous variables, such as age and weight, were assessed for normality using the Shapiro–Wilk test. These variables were reported as median and range, and comparisons between groups were made using the Mann–Whitney U test. Categorical variables were summarized as frequencies and percentages and compared using Fisher’s exact test. All statistical analyses were performed using GraphPad Prism version 10.4.2 (GraphPad Software, USA).

Differences in the dysbiosis index, including all bacterial taxa evaluated by qPCR, and alpha diversity among groups were assessed using the Kruskal–Wallis test when comparing MCT to HC, and the Wilcoxon test when comparing MCT at baseline and at the 30-day follow-up. The Analysis of Similarity (ANOSIM) within the Plymouth Routines In Multivariate Ecological Research (PRIMER-E Ltd., Luton, UK) was used to analyze differences in microbial communities between groups, utilizing Bray–Curtis distance matrices. Analysis of Compositions of Microbiomes with Bias Correction (ANCOM-BC) was used to evaluate changes in the relative abundance of different bacterial taxa.

For the statistical analysis of the immunohistochemistry data, GraphPad Prism version 10.4.2 (GraphPad Software, USA) was used. Differences in the expression of PD-1, PD-L1, and GZMB among dogs with MCT showing complete response (CR), partial response (PR), or progression of disease (PD) were evaluated using the Mann–Whitney test. The Spearman correlation test and Benjamini–Hochberg correction were applied to assess associations between the expression of PD-1, PD-L1, and GZMB and the intestinal microbiota. *p*-values less than 0.05 were considered statistically significant.

## 3. Results

### 3.1. Study Population

Stool samples were collected from 24 healthy client-owned dogs and 24 dogs with cutaneous MCTs (Table 1). All dogs in both groups had hematologic results, kidney parameters, and liver enzyme levels within the normal range and showed no signs of gastrointestinal disease. The MCT group included patients (*n* = 11) diagnosed by fine needle aspiration and cytological examination only. The remaining dogs were diagnosed by tissue biopsy and histological examination and classified as grade I/low-grade MCT (*n* = 2), grade II/low-grade MCT (*n* = 10), and grade II/high-grade MCT (*n* = 1) (Table 3).

Immunohistochemical staining was performed on all available tumor tissue, except for the patient who died immediately after the procedure, to assess the expression of PD-1, PD-L1, and GZMB. Metastases in draining lymph nodes or abdominal organs were not detected or confirmed in any of the patients. All dogs with MCTs underwent ECT and IL-12 GET. Cisplatin was administered to 11 dogs, while bleomycin was used as the chemotherapeutic agent in 13 dogs. The pORFcaIL12ORT plasmid was used in 12 dogs, while the remaining patients received the pCMVcaIL12 plasmid (Table 3).

After four weeks, sixteen dogs showed a complete response, while four dogs had a partial response. Progression of disease was observed in two patients. Owners did not report any significant side effects related to the ECT and IL-12 GET treatment; however, one patient died for unknown reasons immediately after the first treatment, and one dog was euthanized due to neurological symptoms that may have been unrelated to the treatment. Three months after treatment, progression of the disease occurred in five patients, while the remaining patients (*n* = 17) had a complete response.

### 3.2. Canine Dysbiosis Index

**Figure 1:**
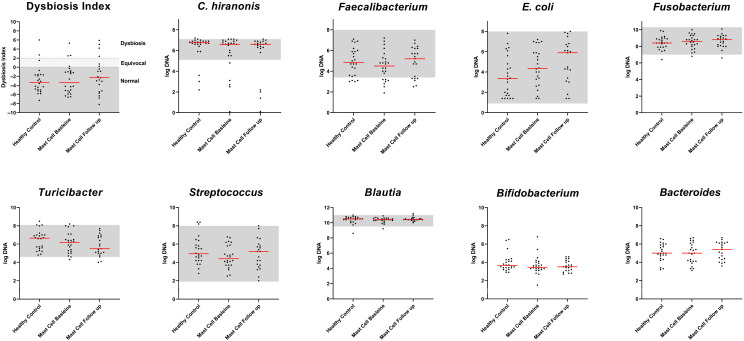
Scatter plots showing the abundances of *Faecalibacterium*, *Turicibacter*, *Streptococcus*, *E. coli*, *Blautia*, *Fusobacterium*, *P. hiranonis*, *Bifidobacterium*, and *Bacteroides*, expressed in log DNA, as well as the dysbiosis index. Data are presented for healthy control dogs and dogs with cutaneous mast cell tumors at baseline and follow-up time points. Note: The gray shaded areas represent the reference intervals for each taxon assessed, and the red lines indicate the median values.

Bacterial abundances and the Canine Dysbiosis Index are presented in Table 4 and Figure 1. There was no significant difference between healthy dogs and dogs with MCTs, nor between baseline and follow-up time points within the MCT group. For DI interpretation, a cut-off value above 2 indicates dysbiosis, and a higher DI means the microbiota deviates further from normal. Values between 0 and 2 are ambiguous and indicate minor shifts in the microbiome, while values below 0 indicate normobiosis.

### 3.3. Species Richness, Diversity, and Evenness

**Figure 2:**
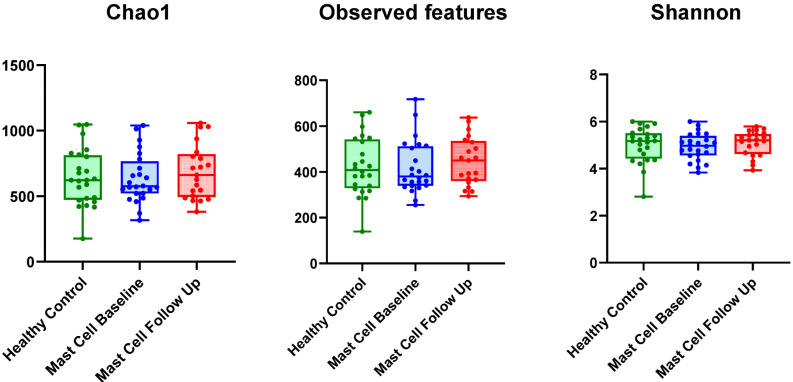
Scatter plots of alpha diversity, including Chao 1, Observed ASVs, and the Shannon Index, for the three groups evaluated in this study: healthy control dogs, baseline samples from patients with cutaneous mast cell tumors (MCT), and follow-up samples from these patients.

Alpha diversity, measured by Chao 1, observed ASVs, and the Shannon Index, did not differ significantly between healthy patients and those with MCT (*p* > 0.05). Similarly, no significant differences in alpha diversity were found when comparing baseline samples to follow-up samples from patients with MCT (*p* > 0.05). Detailed alpha diversity indices are shown in Table 5 and Figure 2.

### 3.4. Microbial Community Structure

**Figure 3:**
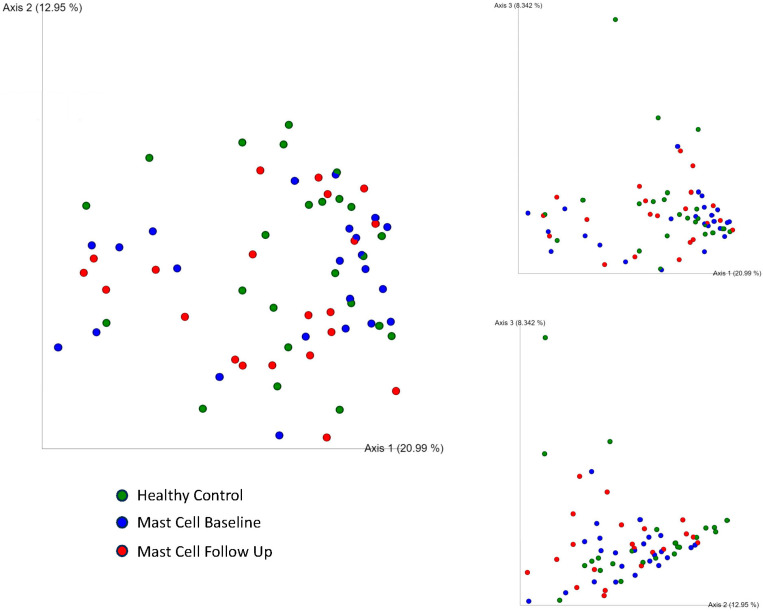
Principal Coordinate Analysis (PCoA) 2D plots of Bray–Curtis distance matrices among healthy controls (green), dogs with cutaneous mast cell tumors sampled at baseline (blue), and at follow-up (red). The PCoA plot of Bray–Curtis distances showed overlapping clustering of microbial communities among healthy controls and MCT groups at both baseline and follow-up. This overlap suggests a similar microbial composition across groups and time points.

Bray–Curtis, a beta diversity measure, showed no significant difference between groups, as indicated by ANOSIM (R = −0.007, *p* = 0.594). Similarly, no significant difference was observed when comparing the intestinal microbial communities of patients at baseline and follow-up time points (R = −0.030, *p* = 0.893). A principal coordinate analysis (PCoA) plot of Bray–Curtis distances showed overlapping clustering of microbial communities among healthy control and MCT groups at both baseline and follow-up time points. This overlap suggests a similar microbial composition across groups and time points (Figure 3 and Supplementary Figure S1).

### 3.5. Microbial Community Composition

**Figure 4:**
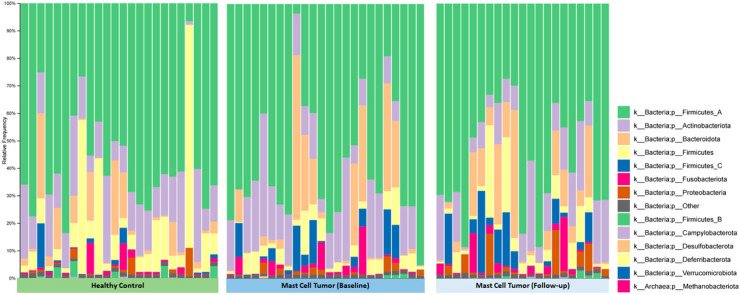
Relative abundance of bacterial taxa annotated at the phylum level for healthy dogs and those with MCTs at baseline and follow-up time points.

Individual bacterial groups were analyzed using ANCOM-BC to compare healthy and MCT groups. At the order level, a significant difference was observed in the abundance of Desulfovibrionales, which increased in the MCT group, while Paenibacillales decreased in the MCT group (*p* < 0.05, Q < 0.05). These taxa were present in exceptionally low abundance. Descriptive statistics, including median and range (minimum and maximum) values for individual taxa, are provided in Supplementary Table S2 and Figure 4.

At the family level, Microbacteriaceae was significantly decreased in the MCT group, while Porphyromonadaceae, Desulfovibrionaceae, and Akkermansiaceae were increased in the MCT group (*p* < 0.05, Q < 0.05). These taxa were present at exceptionally low abundance. At the genus level, Bilophila, Succinivibrio, and Bulleidia were increased in the MCT group. These taxa were also present at exceptionally low abundance.

At the species level, *Bifidobacterium animalis*, *Corynebacterium variabile*, *Lactobacillus johnsonii*, *Pediococcus pentosaceus*, *Streptococcus anginosus*, *Streptococcus equinus*, *Streptococcus intermedius*, *Clostridium thermobutyricum*, *Megasphaera elsdenii*, and *Anaerobiospirillum* sp. were found in lower relative abundance in dogs with MCTs. In contrast, *Bacteroides togonis*, *Lactobacillus amylolyticus*, *Prevotella* sp. CAG:279, and *Megamonas hypermegale* were more abundant in samples from dogs with MCTs (Supplementary Table S3). Descriptive statistics, including median and range (minimum and maximum) values for individual taxa, are provided in Supplementary Table S2.

No major changes were detected using ANCOM-BC to assess shifts in the intestinal microbiota after ECT and IL-12 GET. *Enterococcus gallinarum* increased in follow-up samples from dogs with MCTs, while *Streptococcus gordonii* decreased (*p* < 0.001, Q < 0.01) (Supplementary Table S4). No significant changes were detected using ANCOM-BC to assess shifts in the intestinal microbiota after treatment with bleomycin, cisplatin, pCMVcaIL12 plasmid, or pORFcaIL12ORT plasmid.

### 3.6. PD-1, PD-L1, and GZMB Expression in Cutaneous Mast Cell Tumor

**Figure 5:**
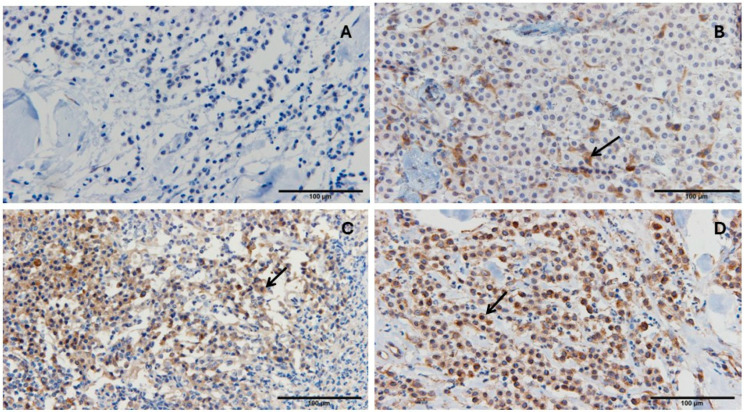
Representative images showing the percentage of immunohistochemically stained cells for PD-1 detection in cutaneous mast cell tumors. (**A**): Cutaneous mast cell tumor with less than 5% stained cells. (**B**): Cutaneous mast cell tumor with 6–25% stained cells. (**C**): Cutaneous mast cell tumor with 26–50% stained cells. (**D**): Cutaneous mast cell tumor with more than 50% stained cells. Immunohistochemical staining was performed using an antibody against PD-1, with hematoxylin counterstaining. Black arrows indicate one representative positively stained cell in each image. The scale bar for 100 µm is shown as a line in each image.

**Figure 6:**
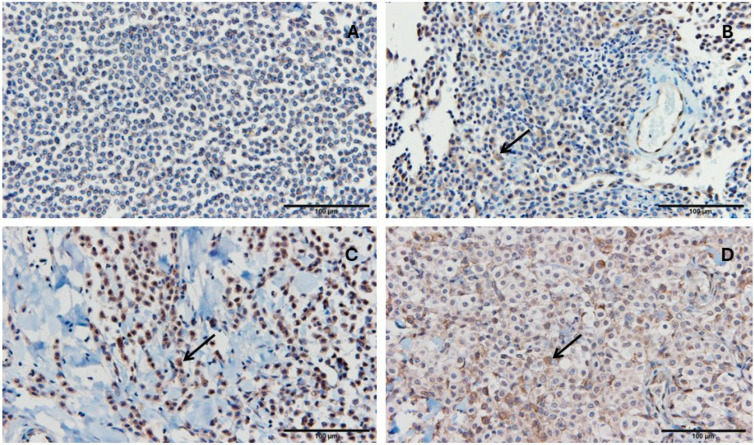
Representative images showing the percentage of immunohistochemically stained cells for PD-L1 detection in cutaneous mast cell tumors. (**A**): Cutaneous mast cell tumor with less than 5% stained cells. (**B**): Cutaneous mast cell tumor with 6–25% stained cells. (**C**): Cutaneous mast cell tumor with 26–50% stained cells. (**D**): Cutaneous mast cell tumor with more than 50% stained cells. Immunohistochemical staining was performed using an antibody against PD-L1, with hematoxylin counterstaining. Black arrows indicate one representative positively stained cell in each image. The scale bar for 100 µm is shown as a line in each image.

**Figure 7:**
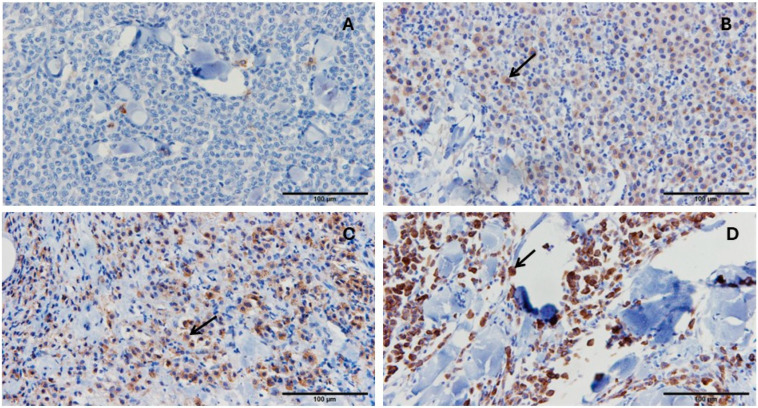
Representative images showing the percentage of immunohistochemically stained cells for Granzyme B detection in cutaneous mast cell tumors. (**A**): Cutaneous mast cell tumor with 5% stained cells. (**B**): Cutaneous mast cell tumor with 30% stained cells. (**C**): Cutaneous mast cell tumor with 50% stained cells. (**D**): Cutaneous mast cell tumor with 75% stained cells. Immunohistochemical staining was performed using an antibody against Granzyme B, with hematoxylin counterstaining. Black arrows indicate one representative positively stained cell in each image. The scale bar for 100 µm is shown as a line in each image.

PD-1 and PD-L1 were evaluated based on the percentage of stained cells and staining intensity. Representative images show the percentage of immunohistochemically stained cells for PD-1 (Figure 5) and PD-L1 (Figure 6). GZMB was analyzed by counting the number of GZMB-positive cells per 100 cells in the captured images. Representative images show the percentage of immunohistochemically stained cells for Granzyme B detection in cutaneous mast cell tumors (Figure 7).

**Figure 8:**
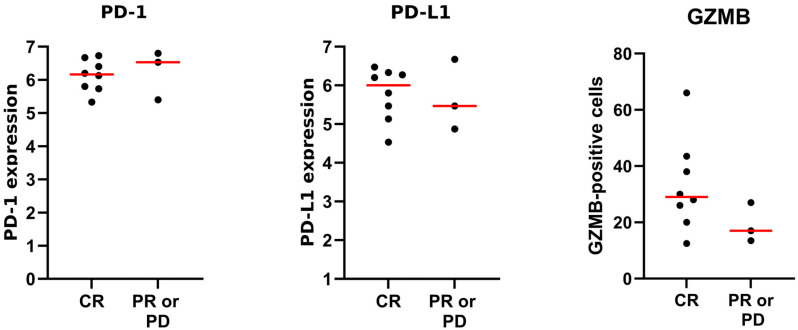
The expression of PD-1, PD-L1, and the number of Granzyme B-positive cells in dogs with MCT was classified based on response to treatment: complete response (CR), partial response (PR), or progression of disease (PD). There was no significant difference between the expression of PD-1, PD-L1, and GZMB and the response to treatment (Mann–Whitney test, *p* > 0.05). Each dot represents an individual patient sample. Red horizontal lines indicate the group median. Note: PD-1: programmed cell death protein 1. PD-L1: programmed cell death 1 ligand 1. GZMB: Granzyme B.

There was no significant difference in the expression of PD-1 and PD-L1 among MCTs. The expression of GZMB-positive cells was significantly higher in some MCTs. We also compared the response to treatment (CR versus PR and PD) in dogs with tumors in terms of PD-1 expression (CR median [range]: 6.2 [5.3–6.7]; PR or PD: 6.5 [5.4–6.8]), PD-L1 (CR: 6.0 [4.5–6.5]; PR or PD: 5.5 [4.9–6.7]), and GZMB (CR: 29.0 [12.5–66.0]; PR or PD: 17 [13.5–27]) (Supplementary Table S5). There was no significant difference in the expression of PD-1, PD-L1, or GZMB between treatment response groups, and no correlation was found between the expression of PD-1, PD-L1, or GZMB and the microbiota (*p* > 0.05) (Figure 8 and Supplementary Table S6).

## 4. Discussion

In this study, we aimed to characterize the fecal microbiota of dogs with MCTs compared to healthy controls and to determine whether significant changes occur after ECT and IL-12 GET treatment, using the dysbiosis index and shallow shotgun metagenomic sequencing. Immunohistochemistry was performed on tumor samples to assess the expression of PD-1, PD-L1, and Granzyme B in cutaneous MCTs. The results indicate that the fecal microbiota of dogs is highly diverse, but no major differences in species richness or bacterial communities were observed in dogs with MCTs compared to healthy controls or after ECT and IL-12 GET treatment. However, an increase in *Enterococcus gallinarum* was detected in follow-up samples from dogs with MCTs, while *Streptococcus gordonii* was reduced.

*Enterococcus gallinarum* is a Gram-positive bacterium that has been shown to be elevated in lupus-prone mice [49]. In germ-free mice mono-colonized with *E. gallinarum*, significant downregulation of gut barrier genes (such as occludin, claudins, and mucin-2) was observed, indicating that this bacterium can weaken gut barrier function and induce a leaky gut. In our study, *E. gallinarum* was increased in follow-up samples from dogs with MCTs; however, gut permeability was not assessed.

Under eubiosis, the most represented phyla in the canine gut microbiome are Actinobacteria, Bacteroidota, Firmicutes, Fusobacteria, and Proteobacteria [50]. This was also observed in our healthy group, with a significantly higher relative abundance of Desulfobacterota in dogs with MCTs compared to healthy dogs.

Although shifts in the relative abundance of certain bacterial taxa were observed, their biological significance should be interpreted with caution. *Bifidobacterium* and *Lactobacillus* species (lactic acid bacteria) are generally considered beneficial members of the gut microbiota because of their roles in supporting the mucosal barrier, modulating the immune system, and producing metabolites such as short-chain fatty acids [51]. Therefore, the lower relative abundance of these taxa in dogs with cutaneous MCTs may be consistent with dysbiosis. Members of the genus *Streptococcus* are also part of the normal canine gut microbiota and have been linked to dysbiosis in some gastrointestinal disorders [52,53]; however, in the present study several *Streptococcus* species were reduced in relative abundance, but these differences were not confirmed by targeted qPCR analysis for either *Streptococcus* or *Bifidobacterium*. This suggests that the apparent changes observed through shotgun sequencing may reflect compositional effects or methodological influences, such as sequencing bias or depth, rather than biologically relevant alterations. Because shotgun sequencing reports taxa as proportions of the total microbial community, shifts in other bacterial groups can produce apparent changes in relative abundance without corresponding changes in the quantity of a given taxon, whereas targeted qPCR provides an independent quantitative measure of specific taxa [54]. Therefore, the functional implications of these microbial shifts remain uncertain without further validation.

Studies have shown a link between dysbiosis and various canine cancers, including mammary cancer, bladder cancer, lymphoma, and melanoma [29,34,55,56]. In both humans and dogs, disease development is influenced by genetic factors, diet, environment, and intestinal microbiota [57,58,59,60,61,62].

A recent study, partially comparable to ours, analyzed the fecal microbiota of healthy dogs and dogs with MCT using 16S rRNA gene sequencing [63]. Although Aluai-Cunha et al. [63] reported differences in alpha diversity based on the inverse Simpson index, which was significantly lower in dogs with MCT, we did not observe any differences in alpha diversity metrics among groups in our study. Similarly, in the analysis of the Bray–Curtis distances, both studies showed considerable overlap between groups (healthy vs. dogs with MCT) [63]. Differences in identified microbial features across studies are a common finding in microbiome research, and in such cases, consistency across studies is more informative than isolated significant findings. Discrepancies between studies are often linked to differences in methodological and statistical approaches, as already mentioned above.

In vivo experiments have further shown that microorganisms play a significant role in carcinogenesis [64,65]. In human medicine, where the role of the microbiota in cancer promotion is being studied more intensively, researchers have found that certain microbes are more abundant in the stool and on the intestinal mucosa of patients with gastrointestinal tumors than in healthy individuals [64,66,67,68]. Certain bacteria, including *Helicobacter pylori*, *E. coli*, *Streptococcus gallolyticus*, *Fusobacterium* sp., and *Bacteroides fragilis*, have been identified in the fecal or tumor samples of human patients with colorectal adenomas and carcinomas [69,70,71,72,73]. However, we did not observe these differences in the dogs with MCT evaluated in our study.

Gastrointestinal complications, including ulcerations that primarily affect the stomach and less commonly the duodenum, are also observed in patients with MCTs, and are much more common in high-grade MCTs [9]. These lesions are usually multiple and superficial, but in some cases, more severe ulceration may develop. This condition is caused by increased levels of histamine in the blood, which stimulate histamine H2 receptors on parietal cells, leading to excessive gastric acid production and increased gastric motility. In addition, histamine damages the vascular endothelium of arterioles and venules and releases fibrinolysin, which promotes intravascular thrombosis and ischemic necrosis of the gastric mucosa [9]. Based on this, we hypothesized that the gut microbiota could also be influenced by the associated inflammation. However, we were unable to confirm such a link. The reasons for this outcome are difficult to determine, but it is possible that peripheral tumors, such as those in the skin or subcutis, may not have a sufficiently prominent systemic effect. Therefore, they may not significantly impact the gut microbiota, but instead have a greater local effect on the skin surface microbiota, as reported previously [74].

Moreover, our study primarily included low-grade MCTs, which typically do not exhibit the previously mentioned systemic effects. This limitation may explain why no significant differences in fecal microbiota composition were observed in these patients compared to healthy individuals. Conducting a future study involving high-grade MCTs would be valuable to better understand the extent to which systemic illness caused by histamine release from high-grade MCTs affects the fecal microbiota and potentially influences treatment outcomes.

Regarding the dysbiosis index, there was no significant difference between healthy dogs and those with MCT, nor between dogs with MCT at baseline and follow-up time points. In contrast, a study comparing the fecal microbiota of healthy dogs and dogs with multicentric malignant B-cell lymphoma found a significantly higher dysbiosis index in the diseased group, suggesting a possible link between the gastrointestinal microbiota and systemic neoplastic diseases [29]. However, it remains unclear whether changes in the microbiota can be associated with the development of cancer. Additionally, the location of cancer and treatment can influence the development of intestinal dysbiosis, highlighting the complexity of this relationship.

In human medicine, there is increasing evidence for the role of the microbiome in immune surveillance, self-tolerance, and response to immune checkpoint inhibitors such as PD-L1 and cytotoxic T-lymphocyte antigen-4 (CTLA-4) blockade [39,75,76,77,78,79]. Immunotherapy targeting immune checkpoints is widely used as a complementary treatment in human cancers but remains poorly explored in veterinary medicine. A recent study on canine cutaneous MCT investigated the expression of the checkpoint proteins PD-L1 and CTLA-4 to evaluate their potential as therapeutic targets [80]. Using immunohistochemistry, the authors analyzed 74 MCT cases and found that most tumors expressed both PD-L1 and CTLA-4 in neoplastic cells. CTLA-4 expression was significantly and inversely associated with tumor grade and mitotic count, while PD-L1 expression was negatively correlated with histological grade and tumor size [80]. This study demonstrates that canine MCT cells express both PD-L1 and CTLA-4, and that their expression is associated with established prognostic factors [80]

Recent studies, including those involving fecal microbiota transplantation (FMT), have shown that modulating the gut microbiome can enhance or inhibit the response to checkpoint immunotherapies [81,82]. As first demonstrated in a mouse model, and more recently, in a human clinical trial, FMT successfully converted a subgroup of non-responding patients into responders, allowing them to benefit from the treatment [39,78,81]. Fecal microbiota from PD-1 responders was transplanted into non-responders. In patients who benefited, the donor bacteria successfully engrafted in the gut and triggered local immune activation, including dendritic-cell stimulation and production of IL-12. This drove a Th1-biased response and boosted cytotoxic CD8^+^ T-cell activity. As a result, tumors developed greater T-cell infiltration and interferon-γ signaling, restoring sensitivity to PD-1 blockade [81]. Many canine cancers, including lymphomas, high-grade gliomas, melanomas, and osteosarcomas, closely resemble their human counterparts, particularly in metastasis, disease recurrence, and response to treatment [79]. Canine patients with naturally occurring cancers also have unaltered immune systems, suggesting that microbiome studies in these patients could provide valuable insights for human medicine, especially in the context of novel immunotherapies that are rapidly advancing in canine comparative oncology [83,84].

While veterinary medicine awaits the widespread commercial availability of canine-specific PD-1, PD-L1, and CTLA-4 monoclonal antibodies to advance immunotherapy in dogs, other immunotherapy approaches are currently in use. Effects on intestinal microbiota and comparisons of microbiome profiles between responder statuses in other immune-based therapies should be considered [79,85,86]. The higher incidence of spontaneous cancers in dogs compared to humans provides valuable insights for developing effective treatment protocols for both species [84,87]. The canine gut microbiome shares 23% homology with the human gut microbiome, compared to only 4.9% with that of mice [79,88], suggesting that the canine model may be more informative than the mouse model in comparative oncology. Unfortunately, our study was unable to establish a link between the expression of PD-1, PD-L1, and Granzyme B in tumor tissue and their effect on the microbiota or treatment response. However, this remains an important area for further research in a larger canine population and across different tumor types, as our study focused solely on cutaneous MCTs.

In our study, all patients with MCT were sampled for the first time under general anesthesia. Although the potential effects of anesthesia on the fecal microbiota should be considered, these effects are likely minimal or negligible in our patients. However, no studies to date have evaluated these effects in dogs or cats. Previous research in rats has shown that exposure to anesthetics can influence the gut microbiome [89]. Some studies have examined the impact of inhalation anesthetics (isoflurane and sevoflurane) and intravenous propofol infusion on the gut microbiome in rats and mice. For example, exposure of rats to the inhalation anesthetic isoflurane can lead to significant changes after four hours of exposure. The composition of the gut microbiome showed significant changes on the first and seventh days after exposure, such as decreased bacterial α-diversity, increased abundances of Proteobacteria and Actinobacteria, and decreased abundances of Firmicutes and Clostridiales [90]. In contrast, a study in rats using 16S rRNA sequencing reported that a three-hour intravenous infusion of propofol had minimal effects on the gut microbiota, with only slight changes observed at the genus level in *Prevotella*, *Lactobacillus*, and *Alloprevotella* [91]. Although current literature suggests significant changes in the gut microbiota following general anesthesia, in our study, fecal samples were collected from dogs with MCT immediately after induction of anesthesia for ECT-GET treatment and before any further procedures. Given the short duration of anesthesia, it can be assumed that the effects on the microbiota would be minimal. Even if there were slight influences, they would have disappeared after one month, when the second sampling was performed.

This study has several important limitations that must be considered when interpreting the findings. The therapy under investigation, electrochemotherapy (ECT) combined with interleukin-12 gene electrotransfer (IL-12 GET), is generally intended for dogs with non-resectable mast cell tumors (MCTs), as well as for cases where surgical resection would result in unacceptable functional impairment or major cosmetic defects. However, some dogs included in this trial had low-grade disease but were enrolled because their owners chose this approach, introducing variability in the treated population. Another significant limitation is that nearly half of the animals were diagnosed exclusively by cytology, which does not allow for tumor grading. While histopathological examination remains the gold standard for definitive diagnosis and grading of MCTs, in the context of a clinical study, this was not always pursued due to financial constraints or owner preferences. Nevertheless, cytological evaluation of the spleen and liver was routinely performed, and additional sampling of the regional lymph nodes was undertaken when ultrasonographic abnormalities were identified, in accordance with current guidelines [41]. Therefore, any signs of systemic metastatic disease would be detected. This issue is particularly relevant for microbiome-related investigations, where precise staging and recognition of systemic disease are crucial, as metastatic involvement has already been shown to influence gut microbial composition in humans [92].

Another limitation is that necropsies were not performed on the two dogs that died during the trial, as the owners declined the procedure for personal reasons. Although both deaths were considered unrelated to treatment based on clinical presentation, the absence of necropsy confirmation prevents definitive conclusions. Necropsy is always recommended to owners, but it is often declined for personal reasons. Based on the clinical presentation, we considered these deaths unrelated to the treatment and therefore retained the animals in the study. Additional variability resulted from including dogs of different ages, as microbiome composition is known to shift with age. To minimize this confounding factor, an age-, sex-, and size-matched healthy control group was established to approximate the MCT cohort, using body size as a proxy in lieu of breed, which could not be standardized. Even with these measures, clinical veterinary studies inherently face limitations, as it is rarely possible to recruit homogeneous groups of animals with identical breed, age, environment, and diet, despite such conditions representing the most controlled scenario. Furthermore, genetic variation, lifestyle factors, and dietary differences inevitably introduce heterogeneity that cannot be fully eliminated. It is already known that different diets have varied impacts on dog microbiota composition [93]. The dogs in our study were not kept in a controlled environment, nor was their diet intake regulated, making it difficult to assess how much these factors affected the microbiota. We also considered which supplements the dogs in our study were taking. Only six dogs from the healthy group and two dogs with cutaneous mast cell tumors were taking vitamin supplements. None of the included dogs received probiotics or additional fiber. There is no specific study in dogs investigating the effect of vitamins on fecal microbiota. However, a human study examined how micronutrient supplementation influences the composition and function of the gut microbiome in healthy adults and concluded that multivitamin/multimineral supplementation did not alter microbiome diversity or structure compared to the control group [94]. We also do not have precise data on the fiber intake of each dog, which could potentially affect the microbiota. In a study conducted in dogs, researchers found that the selection of fiber sources significantly influences host response and gut microbiota [95]. In this study, diets enriched with cereal-based fiber sources led to a more diverse gut ecosystem, resulting in increased alpha diversity and a larger fecal microbial population compared with fruit-based diets [95].

Taken together, these factors highlight the challenges of conducting translational veterinary clinical research, where heterogeneity in diagnosis, staging, patient demographics, and owner-driven decisions must be carefully acknowledged and considered when interpreting study outcomes.

Despite these limitations, this study helps clarify the gut microbiota of healthy dogs and those with MCT, contributing to the current literature, which is expected to expand soon.

## 5. Conclusions

In conclusion, our results indicate that cutaneous MCTs are not associated with fundamental changes in the fecal microbiota, and the ECT and IL-12 GET procedures do not have a notable effect in this regard. We also did not find a link between the expression of PD-1, PD-L1, and GZMB in MCTs and the microbiota, nor any association with treatment response. Further studies with larger patient cohorts are needed to gain a deeper understanding of the role of fecal microbiota in the carcinogenesis of MCTs in dogs. Particular attention should be given to patients with high-grade MCTs, as these cases are more likely to have systemic effects and a significantly greater impact on the microbiota. It is also important to emphasize the safety of ECT and IL-12 GET procedures given their minimal impact on intestinal microbiota and lack of negative systemic effects.

## Figures and Tables

**Table 1:** Demographics of the healthy control group (*n* = 24) and MCT group (*n* = 24), including breed, sex, age, and weight.

	Breed	Sex	Age (Months)	Weight (kg)
**Healthy**	German Pointer	M	86	30
	Giant Schnauzer	F	24	31
	Airedale Terrier	FS	120	26
	Cavalier King Charles spaniel	FS	125	7
	English Cocker Spaniel	FS	140	14
	Tibetan terrier	M	77	12.5
	Whippet	MN	75	17.7
	German Spitz	M	64	6.4
	Lagotto Romagnolo	FS	60	12.5
	Beagle	FS	152	27
	German Shepherd	FS	72	28
	Lagotto Romagnolo	M	109	20
	Russian Greyhound	M	51	42
	Tibetan terrier	M	172	13
	Labrador retriever	F	95	28
	French bulldog	M	94	14
	French bulldog	FS	93	11.8
	French bulldog	FS	88	13
	Keeshond	F	67	16
	German Spitz	M	64	5
	German Spitz	F	115	5
	Pomeranian	F	63	3
	German boxer	M	100	30
	German boxer	M	53	32
**MCT**	Tibetan terrier	F	94	10.8
	German boxer	FS	117	31.8
	Golden retriever	FS	41	33
	French bulldog	F	119	9
	Cross-breed	MC	124	9.3
	Jack Russel Terrier	MC	94	10.3
	Boston terrier	FS	87	8.5
	Cross-breed	FS	135	26
	Swiss mountain dog	FS	40	47.5
	Greyhound	MC	56	31
	Shih-Tzu	MC	78	11
	French bulldog	M	71	9.5
	Mops	F	67	10.8
	Miniature schnauzer	FS	124	7
	Dogo Argentino	FS	83	45
	Dachshund	MC	132	13
	Jack Russel Terrier	FS	142	7
	Cross-breed	F	79	26
	German boxer	M	100	39
	Golden retriever	FS	83	34.6
	Boston terrier	MC	133	116
	Boston terrier	M	73	10
	Newfoundland dog	MC	103	48.5
	Jack Russel Terrier	FS	53	9.8

**Table 2:** Characteristics of the study population: Age and weight are presented as median and range. Sex is reported as the number of animals and the percentage. No statistically significant differences were observed between groups for any variable (*p* > 0.05).

	Healthy Control Group	Mast Cell Tumor Group	*p*-Value
**Age (months)**	87 (24–172)	90 (40–142)	0.6057
**Weight (kg)**	15 (3–42)	11 (7–48)	0.9959
**Sex**			>0.9999
**Female**	54.2% (13/24)	58.3% (14/24)	
**Male**	45.8% (11/24)	41.7% (10/24)	

**Table 3:** Demographics of dogs with cutaneous mast cell tumor (*n* = 24), including breed, sex, age, weight, tumor type and grade, chemotherapeutic used, and plasmid used for each one of the patients included in this study.

Breed	Age (Months)	Sex	Weight (kg)	Tumor Type	Chemotherapeutic Agent	Interleukin IL12 + Plasmid DNA
Miniature schnauzer	124	FS	7	MCT (cytology)	Cisplatin	pCMVcaIL12
Tibetan terrier	94	F	10.8	MCT grade II/low grade	Cisplatin	pCMVcaIL12
German boxer	117	FS	31.8	MCT grade II/low grade	Cisplatin	pORFcaIL12ORT
Golden retriever	41	FS	33	MCT grade II/low grade	Bleomycin	pORFcaIL12ORT
French bulldog	119	F	9	MCT grade II/low grade	Bleomycin	pCMVcaIL12
Dogo Argentino	83	FS	45	MCT (cytology)	Cisplatin	pORFcaIL12ORT
Dachshund	132	MC	13	MCT (cytology)	Bleomycin	pCMVcaIL12
Crossbreed	124	MC	9.3	MCT grade I/low grade	Bleomycin	pCMVcaIL12
Jack Russel Terrier	94	MC	10.3	MCT grade II/low grade	Bleomycin	pCMVcaIL12
Jack Russel Terrier	142	FS	7	MCT (cytology)	Cisplatin	pCMVcaIL12
Crossbreed	79	F	26	MCT (cytology)	Bleomycin	pCMVcaIL12
Boston terrier	87	FS	8.5	MCT grade II/low grade	Cisplatin	pCMVcaIL12
Crossbreed	135	FS	26	MCT grade II/high grade	Bleomycin	pORFcaIL12ORT
German boxer	100	M	39	MCT (cytology)	Cisplatin	pCMVcaIL12
Mops	67	F	10.8	MCT grade II/low grade	Bleomycin	pCMVcaIL12
Golden retriever	83	FS	34.6	MCT (cytology)	Bleomycin	pORFcaIL12ORT
Swiss mountain dog	40	FS	47.5	MCT grade II/low grade	Cisplatin	pCMVcaIL12
Greyhound	56	MC	31	MCT grade I/low grade	Bleomycin	pORFcaIL12ORT
Shih-Tzu	78	MC	11	MCT grade II/low grade	Cisplatin	pORFcaIL12ORT
French bulldog	71	M	9.5	MCT grade II/low grade	Bleomycin	pORFcaIL12ORT
Boston terrier	133	MC	11.6	MCT (cytology)	Bleomycin	pORFcaIL12ORT
Boston terrier	73	M	10	MCT (cytology)	Bleomycin	pORFcaIL12ORT
Newfoundland dog	103	MC	48.5	MCT (cytology)	Cisplatin	pORFcaIL12ORT
Jack Russel Terrier	53	FS	9.8	MCT (cytology)	Cisplatin	pORFcaIL12ORT

**Table 4:** Median and range (min–max) of *Faecalibacterium*, *Turicibacter*, *Streptococcus*, *E. coli*, *Blautia*, *Fusobacterium*, *P. hiranonis*, *Bifidobacterium*, and *Bacteroides* expressed in log DNA, as well as the dysbiosis index, in healthy control dogs and dogs with cutaneous mast cell tumors at baseline and follow-up time points.

	Healthy Control	Mast Cell Tumor (Baseline)	Mast Cell Tumor (Follow-Up)
**Dysbiosis Index**	−3.35 (−7.3 to 6)	−3.4 (−6.6 to 5.3)	−2.3 (−8.2 to 5.9)
***Faecalibacterium***	4.85 (3 to 7.1)	4.5 (1.9 to 7.2)	5.2 (2.5 to 7)
***Turicibacter***	6.65 (4.8 to 8.5)	6.2 (4.3 to 8.2)	5.5 (4 to 7.7)
***Streptococcus***	4.95 (2.8 to 8.4)	4.4 (2.5 to 6.8)	5.2 (2 to 8)
***E. coli***	3.35 (1.4 to 7.8)	4.35 (1.4 to 7.2)	5.9 (1.4 to 8)
***Blautia***	10.5 (8.6 to 11)	10.4 (9.2 to 10.9)	10.4 (10 to 11.2)
***Fusobacterium***	8.4 (6.4 to 9.9)	8.6 (6.8 to 10)	8.8 (6.6 to 10.1)
***P. hiranonis***	6.75 (2.2 to 7.2)	6.6 (0.1 to 7.1)	6.6 (0.1 to 7.1)
***Bifidobacterium***	3.65 (2.9 to 6.5)	3.45 (1.5 to 6.8)	3.5 (2.7 to 4.6)
***Bacteroides***	5 (3.2 to 6.6)	5 (3.1 to 6.7)	5.4 (3.6 to 6.7)

**Table 5:** Summary of alpha diversity indices, including Chao 1, observed ASVs, and the Shannon Index, for the three groups evaluated in this study: healthy control dogs, baseline samples from patients with cutaneous mast cell tumors, and follow-up samples from these patients.

	Healthy Control Group	Mast Cell Tumor (Baseline)	Mast Cell Tumor (Follow-Up)
**Chao 1**	621.9 (176.4–1047)	575.2 (316–1040)	661.4 (381–1059)
**Observed ASVs**	408 (139–660)	379.5 (255–717)	450 (294–637)
**Shannon**	5.172 (2.806–5.998)	4.967 (3.828–6.000)	5.207 (3.925–5.786)

## Data Availability

The original contributions presented in this study are included in the article/Supplementary Material. Further inquiries can be directed to the corresponding author.

## Supplementary Materials

The following supporting information can be downloaded at: https://www.mdpi.com/article/10.3390/vetsci13030241/s1, Supplementary Table S1. Responses from the owners of the included dogs regarding the diet the dog was receiving, vitamins or other supplements, various drugs, and whether the dog was vomiting, had diarrhea, or was experiencing weight loss. Supplementary Table S2. Descriptive statistics, including the median and range (minimum and maximum values), were calculated for the relative abundance of individual taxa, expressed as percentages. Supplementary Table S3. Results of the Analysis of Compositions of Microbiomes with Bias Correction (ANCOM-BC) comparing the gut microbiota of healthy dogs and dogs with cutaneous mast cell tumors (MCT). The table presents the log fold change, *p*-values, and q-values (FDR-adjusted *p*-values) for each taxon, highlighting differences in relative abundance between the two groups. Supplementary Table S4. Results of the Analysis of Compositions of Microbiomes with Bias Correction (ANCOM-BC) comparing the gut microbiota of dogs with cutaneous mast cell tumors at baseline and follow-up time points. The table presents the log fold change, *p*-values, and q-values (FDR-adjusted *p*-values) for each taxon, providing insight into changes in microbial relative abundance following treatment. Supplementary Table S5. PD-1, PD-L1, and GZMB expression in cutaneous MCT patients with response to therapy after 4 weeks and 3 months. Supplementary Table S6. Spearman correlation coefficients (r), *p*-values, and false discovery rate (FDR)-adjusted *p*-values (Benjamini–Hochberg correction) for the association between expression levels of Programmed Cell Death Protein 1 (PD-1), Programmed Death-Ligand 1 (PD-L1), and Granzyme B (GZMB) in tumor tissues and the intestinal microbiota of dogs with cutaneous mast cell tumors. Supplementary Figure S1. Principal Coordinate Analysis (PCoA) 2D plots based on Bray–Curtis distance matrices of fecal microbiota from dogs with cutaneous mast cell tumors at baseline (blue and red) and follow-up (light blue and orange), categorized by treatment response: complete response, partial response, or disease progression. The PCoA revealed overlapping clustering of microbial communities across treatment response groups and time points, suggesting a similar microbial composition regardless of clinical outcome or sampling time.

## Abbreviations

The following abbreviations are used in this manuscript:

MCT	Mast cell tumor
ECT	Electrochemotherapy
IL-12	Interleukin 12
IL-12 GET	Gene electrotransfer of IL-12 plasmid
PD-1	Programmed cell death protein 1
PD-L1	Programmed cell death ligand 1
GZMB	Granzyme B
DI	Dysbiosis index
ICIs	Immune checkpoint inhibitors
CR	Complete response
PR	Partial response
PD	Progression of the disease
