# Supplementary material for: Assessment of Fecal Microbiota in Healthy Dogs and Dogs with Cutaneous Mast Cell Tumors Treated with Electrochemotherapy Combined with Gene Electrotransfer of IL-12
Source: Vet Sci. 2026 Mar 1;13(3):241. doi: 10.3390/vetsci13030241 (PMC13030013; doi:10.3390/vetsci13030241)

Article

Assessment of fecal microbiota in healthy dogs and dogs with cutaneous mast cell tumors treated with electrochemotherapy combined with gene electrotransfer of IL-12

Anja Lisjak[1,*], Bruna Correa Lopes[2], Rachel Pilla[2,3], Ana Nemec[1], Urša Lampreht Tratar[1,4], Jan S. Suchodolski[2] and Nataša Tozon[1]

1 Small Animal Clinic, Veterinary Faculty, University of Ljubljana, Ljubljana, Slovenia

2 Gastrointestinal Laboratory, Department of Small Animal Clinical Sciences, College of Veterinary Medicine & Biomedical Sciences, Texas A&M University, College Station, TX 77843, USA

3 Department of Veterinary Pathology, Hygiene and Public Health, University of Milan, Milan, Italy

4 Department of Experimental Oncology, Institute of Oncology Ljubljana, Ljubljana, Slovenia

* Correspondence: anja.lisjak@vf.uni-lj.si

Supplementary Figure S1. Principal Coordinate Analysis (PCoA) 2D plots based on Bray–Curtis distance matrices of fecal microbiota from dogs with low-grade mast cell tumors at baseline (blue and red) and follow-up (light blue and orange), categorized by treatment response: complete response, partial response, or disease progression. The PCoA revealed overlapping clustering of microbial communities across treatment response groups and time points, suggesting a similar microbial composition regardless of clinical outcome or sampling time.

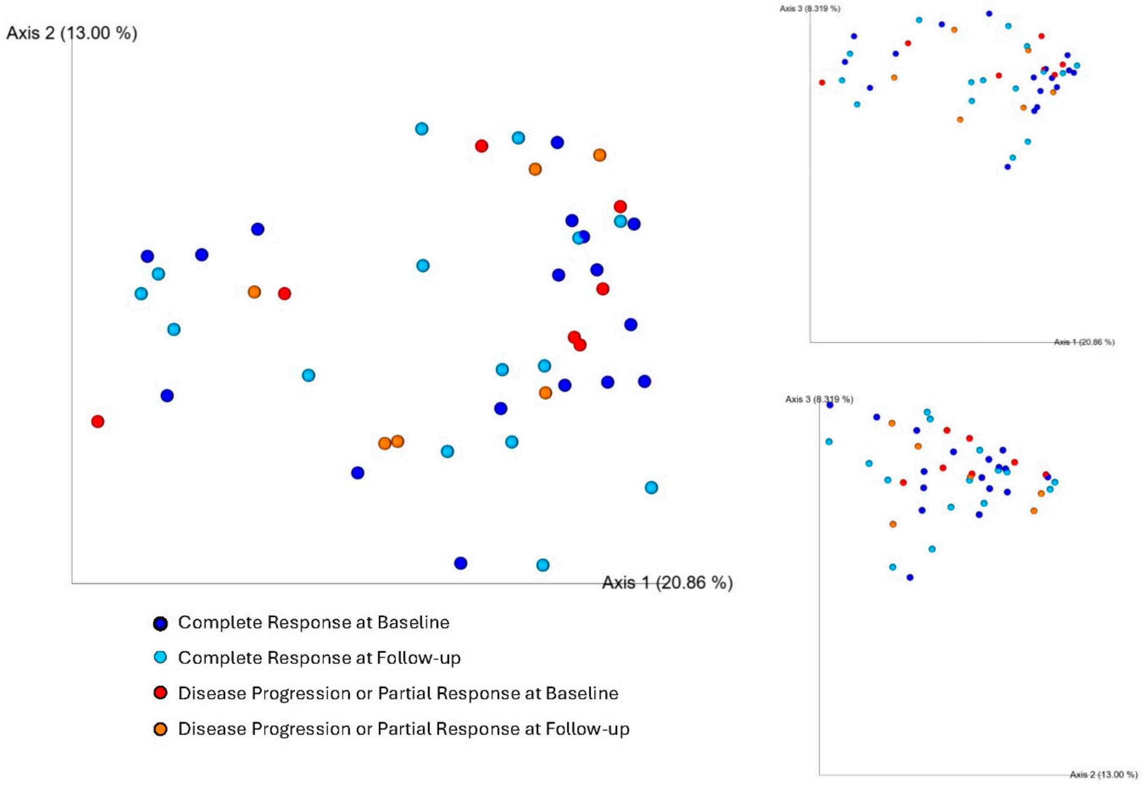

Supplement: Supplementary file 1 [file vetsci-13-00241-s001.zip › vetsci-4127173-supplementary Figure S1.pdf]
