# Supplementary material for: Assessment of Fecal Microbiota in Healthy Dogs and Dogs with Cutaneous Mast Cell Tumors Treated with Electrochemotherapy Combined with Gene Electrotransfer of IL-12
Source: Vet Sci. 2026 Mar 1;13(3):241. doi: 10.3390/vetsci13030241 (PMC13030013; doi:10.3390/vetsci13030241)
Supplement: Supplementary file 1 [file vetsci-13-00241-s001.zip › vetsci-4127173-supplementary Table S1.pdf]

Article

Assessment of fecal microbiota in healthy dogs and dogs with cutaneous mast cell tumors treated with electrochemotherapy combined with gene electrotransfer of IL-12

Anja Lisjak[1,*], Bruna Correa Lopes[2], Rachel Pilla[2,3], Ana Nemec[1], Urša Lampreht Tratar[1,4], Jan S. Suchodolski[2] and Nataša Tozon[1]

1 Small Animal Clinic, Veterinary Faculty, University of Ljubljana, Ljubljana, Slovenia

2 Gastrointestinal Laboratory, Department of Small Animal Clinical Sciences, College of Veterinary Medicine & Biomedical Sciences, Texas A&M University, College Station, TX 77843, USA

3 Department of Veterinary Pathology, Hygiene and Public Health, University of Milan, Milan, Italy

4 Department of Experimental Oncology, Institute of Oncology Ljubljana, Ljubljana, Slovenia

* Correspondence: anja.lisjak@vf.uni-lj.si

Supplementary Table S1. Responses from the owners of the included dogs regarding the diet the dog was receiving, vitamins or other supplements, various drugs, and whether the dog was vomiting, had diarrhea, or was experiencing weight loss.

		Healthy (n=24)	*MCT (n=24)*
Type of food	Dry	12	8
	Wet	0	0
	Dry + Wet	10	6
	Home-cooked	2	2
	Dry + Home-cooked	0	5
	Dry + Wet + Home-cooked	0	3
Treats	Yes	19	21
	No	5	3
Vitamins / Supplements	Yes (vitamins)	6	2
	No	18	22
Antiparasitics	Yes	24	17
	No	0	7
Antibiotics	Yes	0	0
	No	24	24
Immunosuppressives	Yes	0	0
	No	24	24
Gastroprotectants	Yes	0	0
	No	24	24
Vomiting	Yes	0	0
	No	24	24
Diarrhea	Yes	0	0
	No	24	24
Weight loss	Yes	0	0
	No	24	24
