# Supplementary material for: Assessment of Fecal Microbiota in Healthy Dogs and Dogs with Cutaneous Mast Cell Tumors Treated with Electrochemotherapy Combined with Gene Electrotransfer of IL-12
Source: Vet Sci. 2026 Mar 1;13(3):241. doi: 10.3390/vetsci13030241 (PMC13030013; doi:10.3390/vetsci13030241)
Supplement: Supplementary file 1 [file vetsci-13-00241-s001.zip › vetsci-4127173-supplementary Table S5.pdf]

Article

Assessment of fecal microbiota in healthy dogs and dogs with cutaneous mast cell tumors treated with electrochemotherapy combined with gene electrotransfer of IL-12

Anja Lisjak[1,*], Bruna Correa Lopes[2], Rachel Pilla[2,3], Ana Nemec[1], Urša Lampreht Tratar[1,4], Jan S. Suchodolski[2] and Nataša Tozon[1]

1 Small Animal Clinic, Veterinary Faculty, University of Ljubljana, Ljubljana, Slovenia

2 Gastrointestinal Laboratory, Department of Small Animal Clinical Sciences, College of Veterinary Medicine & Biomedical Sciences, Texas A&M University, College Station, TX 77843, USA

3 Department of Veterinary Pathology, Hygiene and Public Health, University of Milan, Milan, Italy

4 Department of Experimental Oncology, Institute of Oncology Ljubljana, Ljubljana, Slovenia

* Correspondence: anja.lisjak@vf.uni-lj.si

Supplementary table S5. PD-1, PD-L1, and GZMB expression in cutaneous MCT patients with response to therapy after 4 weeks and 3 months.

Breed	Age (months)	Sex	Weight (kg)	Tumour type	PD-1	PD-L1	GZMB	Response after 4 weeks	Response after 3 months
Tibetan terrier	94	F	10.8	MCT grade II/low grade	5,4	5,47	13,5	PR	PD
German boxer	117	FS	31.8	MCT grade II/low grade	5,8	5,47	38	CR	CR
Golden retriever	41	FS	33	MCT grade II/low grade	6,2	5,8	12,5	CR	CR
French bulldog	119	F	9	MCT grade II/low grade	6,07	5,33	30	EUTH	
Cross-breed	124	MC	9.3	MCT grade I/low grade	5,33	4,53	43,5	CR	CR
Jack Russel Terrier	94	MC	10.3	MCT grade II/low grade	5,73	6,33	28	CR	CR
Boston terrier	87	FS	8.5	MCT grade II/low grade	6,4	6,47	30	CR	CR
Cross-breed	135	FS	26	MCT grade II/high grade	6,53	4,87	17	PD	PD
Swiss mountain dog	40	FS	47.5	MCT grade II/low grade	6,73	6,27	26	CR	CR
Greyhound	56	MC	31	MCT grade I/low grade	6,8	6,67	27	PR	PD
Shih-Tzu	78	MC	11	MCT grade II/low grade	6,13	5,13	20	CR	CR
French bulldog	71	M	9.5	MCT grade II/low grade	6,67	6,2	66	CR	CR
