# Supplementary material for: Assessment of Fecal Microbiota in Healthy Dogs and Dogs with Cutaneous Mast Cell Tumors Treated with Electrochemotherapy Combined with Gene Electrotransfer of IL-12
Source: Vet Sci. 2026 Mar 1;13(3):241. doi: 10.3390/vetsci13030241 (PMC13030013; doi:10.3390/vetsci13030241)
Supplement: Supplementary file 1 [file vetsci-13-00241-s001.zip › vetsci-4127173-supplementary Tables S2, S3, S4 and S6.pdf]

PDL1vs.k__Bacteria;p__Actinobacteriota
PDL1vs.k__Bacteria;p__Bacteroidota
PDL1vs.k__Bacteria;p__Campylobacterota
PDL1vs.k__Bacteria;p__Firmicutes
PDL1vs.k__Bacteria;p__Firmicutes_A
PDL1vs.k__Bacteria;p__Firmicutes_B
PDL1vs.k__Bacteria;p__Firmicutes_C
PDL1vs.k__Bacteria;p__Fusobacteriota
PDL1vs.k__Bacteria;p__Other
PDL1vs.k__Bacteria;p__Proteobacteria
PDL1vs.k__Bacteria;p__Actinobacteriota;c__Actinomycetia
PDL1vs.k__Bacteria;p__Actinobacteriota;c__Coriobacteriia
PDL1vs.k__Bacteria;p__Bacteroidota;c__Bacteroidia
PDL1vs.k__Bacteria;p__Campylobacterota;c__Campylobacteria
PDL1vs.k__Bacteria;p__Firmicutes;c__Bacilli
PDL1vs.k__Bacteria;p__Firmicutes_A;c__Clostridia
PDL1vs.k__Bacteria;p__Firmicutes_B;c__Peptococcia
PDL1vs.k__Bacteria;p__Firmicutes_C;c__Negativicutes
PDL1vs.k__Bacteria;p__Fusobacteriota;c__Fusobacteriia
PDL1vs.k__Bacteria;p__Other;c__Other
PDL1vs.k__Bacteria;p__Proteobacteria;c__Gammaproteobacteria
PDL1vs.k__Bacteria;p__Actinobacteriota;c__Actinomycetia;o__Actinomycetales
PDL1vs.k__Bacteria;p__Actinobacteriota;c__Actinomycetia;o__Mycobacteriales
PDL1vs.k__Bacteria;p__Actinobacteriota;c__Coriobacteriia;o__Coriobacteriales
PDL1vs.k__Bacteria;p__Bacteroidota;c__Bacteroidia;o__Bacteroidales
PDL1vs.k__Bacteria;p__Campylobacterota;c__Campylobacteria;o__Campylobacterales
PDL1vs.k__Bacteria;p__Firmicutes;c__Bacilli;o__Erysipelotrichales
PDL1vs.k__Bacteria;p__Firmicutes;c__Bacilli;o__Haloplasmatales_A
PDL1vs.k__Bacteria;p__Firmicutes;c__Bacilli;o__Lactobacillales
PDL1vs.k__Bacteria;p__Firmicutes;c__Bacilli;o__Other
PDL1vs.k__Bacteria;p__Firmicutes_A;c__Clostridia;o__Clostridiales
PDL1vs.k__Bacteria;p__Firmicutes_A;c__Clostridia;o__Lachnospirales

PDL1vs.k__Bacteria;p__Firmicutes_A;c__Clostridia;o__Oscillospirales
PDL1vs.k__Bacteria;p__Firmicutes_A;c__Clostridia;o__Other
PDL1vs.k__Bacteria;p__Firmicutes_A;c__Clostridia;o__Peptostreptococcales
PDL1vs.k__Bacteria;p__Firmicutes_B;c__Peptococcia;o__Peptococcales
PDL1vs.k__Bacteria;p__Firmicutes_C;c__Negativicutes;o__Acidaminococcales
PDL1vs.k__Bacteria;p__Firmicutes_C;c__Negativicutes;o__Selenomonadales
PDL1vs.k__Bacteria;p__Fusobacteriota;c__Fusobacteriia;o__Fusobacteriales
PDL1vs.k__Bacteria;p__Other;c__Other;o__Other
PDL1vs.k__Bacteria;p__Proteobacteria;c__Gammaproteobacteria;o__Burkholderiales
PDL1vs.k__Bacteria;p__Proteobacteria;c__Gammaproteobacteria;o__Enterobacterales
PDL1vs.k__Bacteria;p__Actinobacteriota;c__Actinomycetia;o__Actinomycetales;f__Actinomycetaceae
PDL1vs.k__Bacteria;p__Actinobacteriota;c__Actinomycetia;o__Mycobacteriales;f__Mycobacteriaceae
PDL1vs.k__Bacteria;p__Actinobacteriota;c__Coriobacteriia;o__Coriobacteriales;f__Atopobiaceae
PDL1vs.k__Bacteria;p__Actinobacteriota;c__Coriobacteriia;o__Coriobacteriales;f__Coriobacteriaceae
PDL1vs.k__Bacteria;p__Actinobacteriota;c__Coriobacteriia;o__Coriobacteriales;f__Eggerthellaceae
PDL1vs.k__Bacteria;p__Actinobacteriota;c__Coriobacteriia;o__Coriobacteriales;f__Other
PDL1vs.k__Bacteria;p__Bacteroidota;c__Bacteroidia;o__Bacteroidales;f__Bacteroidaceae
PDL1vs.k__Bacteria;p__Bacteroidota;c__Bacteroidia;o__Bacteroidales;f__Muribaculaceae
PDL1vs.k__Bacteria;p__Bacteroidota;c__Bacteroidia;o__Bacteroidales;f__Other
PDL1vs.k__Bacteria;p__Firmicutes;c__Bacilli;o__Erysipelotrichales;f__Erysipelatoclostridiaceae
PDL1vs.k__Bacteria;p__Firmicutes;c__Bacilli;o__Erysipelotrichales;f__Erysipelotrichaceae
PDL1vs.k__Bacteria;p__Firmicutes;c__Bacilli;o__Erysipelotrichales;f__Other
PDL1vs.k__Bacteria;p__Firmicutes;c__Bacilli;o__Haloplasmatales_A;f__Turicibacteraceae
PDL1vs.k__Bacteria;p__Firmicutes;c__Bacilli;o__Lactobacillales;f__Enterococcaceae
PDL1vs.k__Bacteria;p__Firmicutes;c__Bacilli;o__Lactobacillales;f__Lactobacillaceae
PDL1vs.k__Bacteria;p__Firmicutes;c__Bacilli;o__Lactobacillales;f__Streptococcaceae
PDL1vs.k__Bacteria;p__Firmicutes;c__Bacilli;o__Other;f__Other
PDL1vs.k__Bacteria;p__Firmicutes_A;c__Clostridia;o__Clostridiales;f__Clostridiaceae
PDL1vs.k__Bacteria;p__Firmicutes_A;c__Clostridia;o__Lachnospirales;f__Lachnospiraceae
PDL1vs.k__Bacteria;p__Firmicutes_A;c__Clostridia;o__Oscillospirales;f__Butyricicoccaceae
PDL1vs.k__Bacteria;p__Firmicutes_A;c__Clostridia;o__Oscillospirales;f__Oscillospiraceae

PDL1vs.k__Bacteria;p__Firmicutes_A;c__Clostridia;o__Oscillospirales;f__Ruminococcaceae
PDL1vs.k__Bacteria;p__Firmicutes_A;c__Clostridia;o__Other;f__Other
PDL1vs.k__Bacteria;p__Firmicutes_A;c__Clostridia;o__Peptostreptococcales;f__Other
PDL1vs.k__Bacteria;p__Firmicutes_A;c__Clostridia;o__Peptostreptococcales;f__Peptostreptococcaceae
PDL1vs.k__Bacteria;p__Firmicutes_B;c__Peptococcia;o__Peptococcales;f__Peptococcaceae
PDL1vs.k__Bacteria;p__Firmicutes_C;c__Negativicutes;o__Acidaminococcales;f__Acidaminococcaceae
PDL1vs.k__Bacteria;p__Firmicutes_C;c__Negativicutes;o__Selenomonadales;f__Selenomonadaceae
PDL1vs.k__Bacteria;p__Fusobacteriota;c__Fusobacteriia;o__Fusobacteriales;f__Fusobacteriaceae
PDL1vs.k__Bacteria;p__Other;c__Other;o__Other;f__Other
PDL1vs.k__Bacteria;p__Proteobacteria;c__Gammaproteobacteria;o__Burkholderiales;f__Burkholderiaceae
PDL1vs.k__Bacteria;p__Proteobacteria;c__Gammaproteobacteria;o__Enterobacterales;f__Enterobacteriaceae
PDL1vs.k__Bacteria;p__Proteobacteria;c__Gammaproteobacteria;o__Enterobacterales;f__Succinivibrionaceae
PDL1vs.k__Bacteria;p__Actinobacteriota;c__Actinomycetia;o__Actinomycetales;f__Actinomycetaceae;g__Actinomyces
PDL1vs.k__Bacteria;p__Actinobacteriota;c__Actinomycetia;o__Mycobacteriales;f__Mycobacteriaceae;g__Corynebacterium
PDL1vs.k__Bacteria;p__Actinobacteriota;c__Coriobacteriia;o__Coriobacteriales;f__Coriobacteriaceae;g__Collinsella
PDL1vs.k__Bacteria;p__Actinobacteriota;c__Coriobacteriia;o__Coriobacteriales;f__Coriobacteriaceae;g__Other
PDL1vs.k__Bacteria;p__Actinobacteriota;c__Coriobacteriia;o__Coriobacteriales;f__Eggerthellaceae;g__Slackia_A
PDL1vs.k__Bacteria;p__Actinobacteriota;c__Coriobacteriia;o__Coriobacteriales;f__Other;g__Other
PDL1vs.k__Bacteria;p__Bacteroidota;c__Bacteroidia;o__Bacteroidales;f__Bacteroidaceae;g__Bacteroides
PDL1vs.k__Bacteria;p__Bacteroidota;c__Bacteroidia;o__Bacteroidales;f__Bacteroidaceae;g__Other
PDL1vs.k__Bacteria;p__Bacteroidota;c__Bacteroidia;o__Bacteroidales;f__Bacteroidaceae;g__Paraprevotella
PDL1vs.k__Bacteria;p__Bacteroidota;c__Bacteroidia;o__Bacteroidales;f__Bacteroidaceae;g__Phocaeicola
PDL1vs.k__Bacteria;p__Bacteroidota;c__Bacteroidia;o__Bacteroidales;f__Bacteroidaceae;g__Prevotella
PDL1vs.k__Bacteria;p__Bacteroidota;c__Bacteroidia;o__Bacteroidales;f__Bacteroidaceae;g__Prevotellamassilia
PDL1vs.k__Bacteria;p__Bacteroidota;c__Bacteroidia;o__Bacteroidales;f__Muribaculaceae;g__CAG-279
PDL1vs.k__Bacteria;p__Bacteroidota;c__Bacteroidia;o__Bacteroidales;f__Other;g__Other
PDL1vs.k__Bacteria;p__Firmicutes;c__Bacilli;o__Erysipelotrichales;f__Erysipelatoclostridiaceae;g__Catenibacterium
PDL1vs.k__Bacteria;p__Firmicutes;c__Bacilli;o__Erysipelotrichales;f__Erysipelatoclostridiaceae;g__Erysipelatoclostridium
PDL1vs.k__Bacteria;p__Firmicutes;c__Bacilli;o__Erysipelotrichales;f__Erysipelatoclostridiaceae;g__Faecalibacillus
PDL1vs.k__Bacteria;p__Firmicutes;c__Bacilli;o__Erysipelotrichales;f__Erysipelatoclostridiaceae;g__Other
PDL1vs.k__Bacteria;p__Firmicutes;c__Bacilli;o__Erysipelotrichales;f__Erysipelatoclostridiaceae;g__[Erysipelatoclostridiaceae_Garnier_p1_[illegible]

PDL1vs.k__Bacteria;p__Firmicutes;c__Bacilli;o__Erysipelotrichales;f__Erysipelatoclostridiaceae;g__[Erysipelatoclostridiaceae_Snoopy_p2_
PDL1vs.k__Bacteria;p__Firmicutes;c__Bacilli;o__Erysipelotrichales;f__Erysipelotrichaceae;g__Allobaculum
PDL1vs.k__Bacteria;p__Firmicutes;c__Bacilli;o__Erysipelotrichales;f__Erysipelotrichaceae;g__Amedibacillus
PDL1vs.k__Bacteria;p__Firmicutes;c__Bacilli;o__Erysipelotrichales;f__Erysipelotrichaceae;g__Amedibacterium
PDL1vs.k__Bacteria;p__Firmicutes;c__Bacilli;o__Erysipelotrichales;f__Erysipelotrichaceae;g__Clostridium_AQ
PDL1vs.k__Bacteria;p__Firmicutes;c__Bacilli;o__Erysipelotrichales;f__Erysipelotrichaceae;g__Holdemanella
PDL1vs.k__Bacteria;p__Firmicutes;c__Bacilli;o__Erysipelotrichales;f__Erysipelotrichaceae;g__Holdemania
PDL1vs.k__Bacteria;p__Firmicutes;c__Bacilli;o__Erysipelotrichales;f__Erysipelotrichaceae;g__Other
PDL1vs.k__Bacteria;p__Firmicutes;c__Bacilli;o__Erysipelotrichales;f__Erysipelotrichaceae;g__[Erysipelotrichaceae_Scrappy_p2_maxbin2_l
PDL1vs.k__Bacteria;p__Firmicutes;c__Bacilli;o__Erysipelotrichales;f__Other;g__Other
PDL1vs.k__Bacteria;p__Firmicutes;c__Bacilli;o__Haloplasmatales_A;f__Turicibacteraceae;g__Turicibacter
PDL1vs.k__Bacteria;p__Firmicutes;c__Bacilli;o__Lactobacillales;f__Lactobacillaceae;g__Lactobacillus
PDL1vs.k__Bacteria;p__Firmicutes;c__Bacilli;o__Lactobacillales;f__Lactobacillaceae;g__Latilactobacillus
PDL1vs.k__Bacteria;p__Firmicutes;c__Bacilli;o__Lactobacillales;f__Lactobacillaceae;g__Leuconostoc
PDL1vs.k__Bacteria;p__Firmicutes;c__Bacilli;o__Lactobacillales;f__Lactobacillaceae;g__Other
PDL1vs.k__Bacteria;p__Firmicutes;c__Bacilli;o__Lactobacillales;f__Streptococcaceae;g__Lactococcus
PDL1vs.k__Bacteria;p__Firmicutes;c__Bacilli;o__Lactobacillales;f__Streptococcaceae;g__Streptococcus
PDL1vs.k__Bacteria;p__Firmicutes;c__Bacilli;o__Other;f__Other;g__Other
PDL1vs.k__Bacteria;p__Firmicutes_A;c__Clostridia;o__Clostridiales;f__Clostridiaceae;g__Clostridium
PDL1vs.k__Bacteria;p__Firmicutes_A;c__Clostridia;o__Clostridiales;f__Clostridiaceae;g__Clostridium_P
PDL1vs.k__Bacteria;p__Firmicutes_A;c__Clostridia;o__Clostridiales;f__Clostridiaceae;g__Other
PDL1vs.k__Bacteria;p__Firmicutes_A;c__Clostridia;o__Lachnospirales;f__Lachnospiraceae;g__Acetatifactor
PDL1vs.k__Bacteria;p__Firmicutes_A;c__Clostridia;o__Lachnospirales;f__Lachnospiraceae;g__Anaerostipes
PDL1vs.k__Bacteria;p__Firmicutes_A;c__Clostridia;o__Lachnospirales;f__Lachnospiraceae;g__Bariatricus
PDL1vs.k__Bacteria;p__Firmicutes_A;c__Clostridia;o__Lachnospirales;f__Lachnospiraceae;g__Blautia
PDL1vs.k__Bacteria;p__Firmicutes_A;c__Clostridia;o__Lachnospirales;f__Lachnospiraceae;g__Blautia_A
PDL1vs.k__Bacteria;p__Firmicutes_A;c__Clostridia;o__Lachnospirales;f__Lachnospiraceae;g__CAG-81
PDL1vs.k__Bacteria;p__Firmicutes_A;c__Clostridia;o__Lachnospirales;f__Lachnospiraceae;g__Clostridium_Q
PDL1vs.k__Bacteria;p__Firmicutes_A;c__Clostridia;o__Lachnospirales;f__Lachnospiraceae;g__Coprococcus
PDL1vs.k__Bacteria;p__Firmicutes_A;c__Clostridia;o__Lachnospirales;f__Lachnospiraceae;g__Dorea
PDL1vs.k__Bacteria;p__Firmicutes_A;c__Clostridia;o__Lachnospirales;f__Lachnospiraceae;g__Dorea_A

PDL1vs.k__Bacteria;p__Firmicutes_A;c__Clostridia;o__Lachnospirales;f__Lachnospiraceae;g__Dorea_B
PDL1vs.k__Bacteria;p__Firmicutes_A;c__Clostridia;o__Lachnospirales;f__Lachnospiraceae;g__Eisenbergiella
PDL1vs.k__Bacteria;p__Firmicutes_A;c__Clostridia;o__Lachnospirales;f__Lachnospiraceae;g__Enterocloster
PDL1vs.k__Bacteria;p__Firmicutes_A;c__Clostridia;o__Lachnospirales;f__Lachnospiraceae;g__Eubacterium_I
PDL1vs.k__Bacteria;p__Firmicutes_A;c__Clostridia;o__Lachnospirales;f__Lachnospiraceae;g__Faecalimonas
PDL1vs.k__Bacteria;p__Firmicutes_A;c__Clostridia;o__Lachnospirales;f__Lachnospiraceae;g__Fusicatenibacter
PDL1vs.k__Bacteria;p__Firmicutes_A;c__Clostridia;o__Lachnospirales;f__Lachnospiraceae;g__Lachnospira
PDL1vs.k__Bacteria;p__Firmicutes_A;c__Clostridia;o__Lachnospirales;f__Lachnospiraceae;g__Mediterraneibacter
PDL1vs.k__Bacteria;p__Firmicutes_A;c__Clostridia;o__Lachnospirales;f__Lachnospiraceae;g__Other
PDL1vs.k__Bacteria;p__Firmicutes_A;c__Clostridia;o__Lachnospirales;f__Lachnospiraceae;g__Roseburia
PDL1vs.k__Bacteria;p__Firmicutes_A;c__Clostridia;o__Lachnospirales;f__Lachnospiraceae;g__Ruminococcus_A
PDL1vs.k__Bacteria;p__Firmicutes_A;c__Clostridia;o__Lachnospirales;f__Lachnospiraceae;g__Ruminococcus_B
PDL1vs.k__Bacteria;p__Firmicutes_A;c__Clostridia;o__Lachnospirales;f__Lachnospiraceae;g__Schaedlerella
PDL1vs.k__Bacteria;p__Firmicutes_A;c__Clostridia;o__Lachnospirales;f__Lachnospiraceae;g__UBA9502
PDL1vs.k__Bacteria;p__Firmicutes_A;c__Clostridia;o__Oscillospirales;f__Butyricicoccaceae;g__Agathobaculum
PDL1vs.k__Bacteria;p__Firmicutes_A;c__Clostridia;o__Oscillospirales;f__Butyricicoccaceae;g__Butyricicoccus
PDL1vs.k__Bacteria;p__Firmicutes_A;c__Clostridia;o__Oscillospirales;f__Oscillospiraceae;g__CAG-110
PDL1vs.k__Bacteria;p__Firmicutes_A;c__Clostridia;o__Oscillospirales;f__Oscillospiraceae;g__Flavonifractor
PDL1vs.k__Bacteria;p__Firmicutes_A;c__Clostridia;o__Oscillospirales;f__Ruminococcaceae;g__Faecalibacterium
PDL1vs.k__Bacteria;p__Firmicutes_A;c__Clostridia;o__Oscillospirales;f__Ruminococcaceae;g__Fournierella
PDL1vs.k__Bacteria;p__Firmicutes_A;c__Clostridia;o__Oscillospirales;f__Ruminococcaceae;g__Negativibacillus
PDL1vs.k__Bacteria;p__Firmicutes_A;c__Clostridia;o__Oscillospirales;f__Ruminococcaceae;g__UMGS966
PDL1vs.k__Bacteria;p__Firmicutes_A;c__Clostridia;o__Other;f__Other;g__Other
PDL1vs.k__Bacteria;p__Firmicutes_A;c__Clostridia;o__Peptostreptococcales;f__Other;g__Other
PDL1vs.k__Bacteria;p__Firmicutes_A;c__Clostridia;o__Peptostreptococcales;f__Peptostreptococcaceae;g__Clostridioides
PDL1vs.k__Bacteria;p__Firmicutes_A;c__Clostridia;o__Peptostreptococcales;f__Peptostreptococcaceae;g__Other
PDL1vs.k__Bacteria;p__Firmicutes_A;c__Clostridia;o__Peptostreptococcales;f__Peptostreptococcaceae;g__Paraclostridium
PDL1vs.k__Bacteria;p__Firmicutes_A;c__Clostridia;o__Peptostreptococcales;f__Peptostreptococcaceae;g__Peptacetobacter
PDL1vs.k__Bacteria;p__Firmicutes_A;c__Clostridia;o__Peptostreptococcales;f__Peptostreptococcaceae;g__Peptostreptococcus
PDL1vs.k__Bacteria;p__Firmicutes_A;c__Clostridia;o__Peptostreptococcales;f__Peptostreptococcaceae;g__Romboutsia
PDL1vs.k__Bacteria;p__Firmicutes_A;c__Clostridia;o__Peptostreptococcales;f__Peptostreptococcaceae;g__Terrisporobacter

PDL1vs.k__Bacteria;p__Firmicutes_B;c__Peptococcia;o__Peptococcales;f__Peptococcaceae;g__UMGS1590
PDL1vs.k__Bacteria;p__Firmicutes_C;c__Negativicutes;o__Acidaminococcales;f__Acidaminococcaceae;g__Phascolarctobacterium_A
PDL1vs.k__Bacteria;p__Firmicutes_C;c__Negativicutes;o__Selenomonadales;f__Selenomonadaceae;g__Megamonas
PDL1vs.k__Bacteria;p__Fusobacteriota;c__Fusobacteriia;o__Fusobacteriales;f__Fusobacteriaceae;g__Fusobacterium_A
PDL1vs.k__Bacteria;p__Fusobacteriota;c__Fusobacteriia;o__Fusobacteriales;f__Fusobacteriaceae;g__Fusobacterium_B
PDL1vs.k__Bacteria;p__Fusobacteriota;c__Fusobacteriia;o__Fusobacteriales;f__Fusobacteriaceae;g__Other
PDL1vs.k__Bacteria;p__Other;c__Other;o__Other;f__Other;g__Other
PDL1vs.k__Bacteria;p__Proteobacteria;c__Gammaproteobacteria;o__Burkholderiales;f__Burkholderiaceae;g__Sutterella
PDL1vs.k__Bacteria;p__Proteobacteria;c__Gammaproteobacteria;o__Enterobacterales;f__Enterobacteriaceae;g__Escherichia
PDL1vs.k__Bacteria;p__Proteobacteria;c__Gammaproteobacteria;o__Enterobacterales;f__Enterobacteriaceae;g__Other
PDL1vs.k__Bacteria;p__Actinobacteriota;c__Coriobacteriia;o__Coriobacteriales;f__Coriobacteriaceae;g__Collinsella;s__Collinsella_intestina
PDL1vs.k__Bacteria;p__Actinobacteriota;c__Coriobacteriia;o__Coriobacteriales;f__Coriobacteriaceae;g__Collinsella;s__Collinsella_phocaee
PDL1vs.k__Bacteria;p__Actinobacteriota;c__Coriobacteriia;o__Coriobacteriales;f__Coriobacteriaceae;g__Collinsella;s__Collinsella_stercoris
PDL1vs.k__Bacteria;p__Actinobacteriota;c__Coriobacteriia;o__Coriobacteriales;f__Coriobacteriaceae;g__Collinsella;s__Collinsella_tanakaei
PDL1vs.k__Bacteria;p__Actinobacteriota;c__Coriobacteriia;o__Coriobacteriales;f__Coriobacteriaceae;g__Collinsella;s__Other
PDL1vs.k__Bacteria;p__Actinobacteriota;c__Coriobacteriia;o__Coriobacteriales;f__Coriobacteriaceae;g__Collinsella;s__[Collinsella_X13_Wo
PDL1vs.k__Bacteria;p__Actinobacteriota;c__Coriobacteriia;o__Coriobacteriales;f__Coriobacteriaceae;g__Other;s__Other
PDL1vs.k__Bacteria;p__Actinobacteriota;c__Coriobacteriia;o__Coriobacteriales;f__Eggerthellaceae;g__Slackia_A;s__Slackia_A_faecicanis
PDL1vs.k__Bacteria;p__Actinobacteriota;c__Coriobacteriia;o__Coriobacteriales;f__Eggerthellaceae;g__Slackia_A;s__Slackia_A_piriformis
PDL1vs.k__Bacteria;p__Actinobacteriota;c__Coriobacteriia;o__Coriobacteriales;f__Other;g__Other;s__Other
PDL1vs.k__Bacteria;p__Bacteroidota;c__Bacteroidia;o__Bacteroidales;f__Bacteroidaceae;g__Bacteroides;s__Bacteroides_fluxus
PDL1vs.k__Bacteria;p__Bacteroidota;c__Bacteroidia;o__Bacteroidales;f__Bacteroidaceae;g__Bacteroides;s__Bacteroides_sp900766005
PDL1vs.k__Bacteria;p__Bacteroidota;c__Bacteroidia;o__Bacteroidales;f__Bacteroidaceae;g__Bacteroides;s__Other
PDL1vs.k__Bacteria;p__Bacteroidota;c__Bacteroidia;o__Bacteroidales;f__Bacteroidaceae;g__Other;s__Other
PDL1vs.k__Bacteria;p__Bacteroidota;c__Bacteroidia;o__Bacteroidales;f__Bacteroidaceae;g__Phocaeicola;s__Other
PDL1vs.k__Bacteria;p__Bacteroidota;c__Bacteroidia;o__Bacteroidales;f__Bacteroidaceae;g__Phocaeicola;s__Phocaeicola_coprocola
PDL1vs.k__Bacteria;p__Bacteroidota;c__Bacteroidia;o__Bacteroidales;f__Bacteroidaceae;g__Phocaeicola;s__Phocaeicola_plebeius
PDL1vs.k__Bacteria;p__Bacteroidota;c__Bacteroidia;o__Bacteroidales;f__Bacteroidaceae;g__Phocaeicola;s__Phocaeicola_sp900546645
PDL1vs.k__Bacteria;p__Bacteroidota;c__Bacteroidia;o__Bacteroidales;f__Bacteroidaceae;g__Phocaeicola;s__Phocaeicola_vulgatus
PDL1vs.k__Bacteria;p__Bacteroidota;c__Bacteroidia;o__Bacteroidales;f__Bacteroidaceae;g__Prevotella;s__Other
PDL1vs.k__Bacteria;p__Bacteroidota;c__Bacteroidia;o__Bacteroidales;f__Bacteroidaceae;g__Prevotella;s__Prevotella_copri

PDL1vs.k__Bacteria;p__Bacteroidota;c__Bacteroidia;o__Bacteroidales;f__Bacteroidaceae;g__Prevotellamassilia;s__Other
PDL1vs.k__Bacteria;p__Bacteroidota;c__Bacteroidia;o__Bacteroidales;f__Bacteroidaceae;g__Prevotellamassilia;s__Prevotellamassilia_sp00
PDL1vs.k__Bacteria;p__Bacteroidota;c__Bacteroidia;o__Bacteroidales;f__Muribaculaceae;g__CAG-279;s__CAG-279_sp900541935
PDL1vs.k__Bacteria;p__Bacteroidota;c__Bacteroidia;o__Bacteroidales;f__Other;g__Other;s__Other
PDL1vs.k__Bacteria;p__Firmicutes;c__Bacilli;o__Erysipelotrichales;f__Erysipelatoclostridiaceae;g__Catenibacterium;s__Catenibacterium_s
PDL1vs.k__Bacteria;p__Firmicutes;c__Bacilli;o__Erysipelotrichales;f__Erysipelatoclostridiaceae;g__Erysipelatoclostridium;s__Erysipelatocl
PDL1vs.k__Bacteria;p__Firmicutes;c__Bacilli;o__Erysipelotrichales;f__Erysipelatoclostridiaceae;g__Erysipelatoclostridium;s__Erysipelatocl
PDL1vs.k__Bacteria;p__Firmicutes;c__Bacilli;o__Erysipelotrichales;f__Erysipelatoclostridiaceae;g__Erysipelatoclostridium;s__Other
PDL1vs.k__Bacteria;p__Firmicutes;c__Bacilli;o__Erysipelotrichales;f__Erysipelatoclostridiaceae;g__Erysipelatoclostridium;s__[Erysipelatoc
PDL1vs.k__Bacteria;p__Firmicutes;c__Bacilli;o__Erysipelotrichales;f__Erysipelatoclostridiaceae;g__Faecalibacillus;s__Faecalibacillus_intes
PDL1vs.k__Bacteria;p__Firmicutes;c__Bacilli;o__Erysipelotrichales;f__Erysipelatoclostridiaceae;g__Other;s__Other
PDL1vs.k__Bacteria;p__Firmicutes;c__Bacilli;o__Erysipelotrichales;f__Erysipelatoclostridiaceae;g__[Erysipelatoclostridiaceae_Garnier_p1_
PDL1vs.k__Bacteria;p__Firmicutes;c__Bacilli;o__Erysipelotrichales;f__Erysipelatoclostridiaceae;g__[Erysipelatoclostridiaceae_Snoopy_p2_
PDL1vs.k__Bacteria;p__Firmicutes;c__Bacilli;o__Erysipelotrichales;f__Erysipelotrichaceae;g__Allobaculum;s__Allobaculum_stercoricanis
PDL1vs.k__Bacteria;p__Firmicutes;c__Bacilli;o__Erysipelotrichales;f__Erysipelotrichaceae;g__Amedibacillus;s__Amedibacillus_dolichus
PDL1vs.k__Bacteria;p__Firmicutes;c__Bacilli;o__Erysipelotrichales;f__Erysipelotrichaceae;g__Amedibacterium;s__Amedibacterium_intestin
PDL1vs.k__Bacteria;p__Firmicutes;c__Bacilli;o__Erysipelotrichales;f__Erysipelotrichaceae;g__Clostridium_AQ;s__Clostridium_AQ_innocuu
PDL1vs.k__Bacteria;p__Firmicutes;c__Bacilli;o__Erysipelotrichales;f__Erysipelotrichaceae;g__Holdemanella;s__Holdemanella_sp002299315
PDL1vs.k__Bacteria;p__Firmicutes;c__Bacilli;o__Erysipelotrichales;f__Erysipelotrichaceae;g__Other;s__Other
PDL1vs.k__Bacteria;p__Firmicutes;c__Bacilli;o__Erysipelotrichales;f__Erysipelotrichaceae;g__[Erysipelotrichaceae_Scrappy_p2_maxbin2_l
PDL1vs.k__Bacteria;p__Firmicutes;c__Bacilli;o__Erysipelotrichales;f__Other;g__Other;s__Other
PDL1vs.k__Bacteria;p__Firmicutes;c__Bacilli;o__Haloplasmatales_A;f__Turicibacteraceae;g__Turicibacter;s__Turicibacter_sp001543345
PDL1vs.k__Bacteria;p__Firmicutes;c__Bacilli;o__Haloplasmatales_A;f__Turicibacteraceae;g__Turicibacter;s__Turicibacter_sp002311155
PDL1vs.k__Bacteria;p__Firmicutes;c__Bacilli;o__Lactobacillales;f__Lactobacillaceae;g__Latilactobacillus;s__Latilactobacillus_curvatus
PDL1vs.k__Bacteria;p__Firmicutes;c__Bacilli;o__Lactobacillales;f__Lactobacillaceae;g__Other;s__Other
PDL1vs.k__Bacteria;p__Firmicutes;c__Bacilli;o__Lactobacillales;f__Streptococcaceae;g__Streptococcus;s__Other
PDL1vs.k__Bacteria;p__Firmicutes;c__Bacilli;o__Other;f__Other;g__Other;s__Other
PDL1vs.k__Bacteria;p__Firmicutes_A;c__Clostridia;o__Clostridiales;f__Clostridiaceae;g__Clostridium;s__Clostridium_paraputrificum
PDL1vs.k__Bacteria;p__Firmicutes_A;c__Clostridia;o__Clostridiales;f__Clostridiaceae;g__Clostridium_P;s__Clostridium_P_perfringens
PDL1vs.k__Bacteria;p__Firmicutes_A;c__Clostridia;o__Clostridiales;f__Clostridiaceae;g__Other;s__Other
PDL1vs.k__Bacteria;p__Firmicutes_A;c__Clostridia;o__Lachnospirales;f__Lachnospiraceae;g__Acetatifactor;s__Acetatifactor_sp900066565

PDL1vs.k__Bacteria;p__Firmicutes_A;c__Clostridia;o__Lachnospirales;f__Lachnospiraceae;g__Bariatricus;s__Bariatricus_comes
PDL1vs.k__Bacteria;p__Firmicutes_A;c__Clostridia;o__Lachnospirales;f__Lachnospiraceae;g__Blautia;s__Blautia_coccoides
PDL1vs.k__Bacteria;p__Firmicutes_A;c__Clostridia;o__Lachnospirales;f__Lachnospiraceae;g__Blautia;s__Blautia_hansenii
PDL1vs.k__Bacteria;p__Firmicutes_A;c__Clostridia;o__Lachnospirales;f__Lachnospiraceae;g__Blautia;s__Blautia_sp002161285
PDL1vs.k__Bacteria;p__Firmicutes_A;c__Clostridia;o__Lachnospirales;f__Lachnospiraceae;g__Blautia;s__Blautia_sp003287895
PDL1vs.k__Bacteria;p__Firmicutes_A;c__Clostridia;o__Lachnospirales;f__Lachnospiraceae;g__Blautia;s__Blautia_sp900120295
PDL1vs.k__Bacteria;p__Firmicutes_A;c__Clostridia;o__Lachnospirales;f__Lachnospiraceae;g__Blautia;s__Blautia_sp900556555
PDL1vs.k__Bacteria;p__Firmicutes_A;c__Clostridia;o__Lachnospirales;f__Lachnospiraceae;g__Blautia;s__Blautia_stercoris
PDL1vs.k__Bacteria;p__Firmicutes_A;c__Clostridia;o__Lachnospirales;f__Lachnospiraceae;g__Blautia;s__Other
PDL1vs.k__Bacteria;p__Firmicutes_A;c__Clostridia;o__Lachnospirales;f__Lachnospiraceae;g__Blautia_A;s__Blautia_A_caecimuris
PDL1vs.k__Bacteria;p__Firmicutes_A;c__Clostridia;o__Lachnospirales;f__Lachnospiraceae;g__Blautia_A;s__Blautia_A_faecis
PDL1vs.k__Bacteria;p__Firmicutes_A;c__Clostridia;o__Lachnospirales;f__Lachnospiraceae;g__Blautia_A;s__Blautia_A_massiliensis
PDL1vs.k__Bacteria;p__Firmicutes_A;c__Clostridia;o__Lachnospirales;f__Lachnospiraceae;g__Blautia_A;s__Blautia_A_obeum
PDL1vs.k__Bacteria;p__Firmicutes_A;c__Clostridia;o__Lachnospirales;f__Lachnospiraceae;g__Blautia_A;s__Blautia_A_sp900066145
PDL1vs.k__Bacteria;p__Firmicutes_A;c__Clostridia;o__Lachnospirales;f__Lachnospiraceae;g__Blautia_A;s__Blautia_A_sp900541345
PDL1vs.k__Bacteria;p__Firmicutes_A;c__Clostridia;o__Lachnospirales;f__Lachnospiraceae;g__Blautia_A;s__Blautia_A_wexlerae
PDL1vs.k__Bacteria;p__Firmicutes_A;c__Clostridia;o__Lachnospirales;f__Lachnospiraceae;g__Blautia_A;s__Blautia_A_wexlerae_A
PDL1vs.k__Bacteria;p__Firmicutes_A;c__Clostridia;o__Lachnospirales;f__Lachnospiraceae;g__Blautia_A;s__Other
PDL1vs.k__Bacteria;p__Firmicutes_A;c__Clostridia;o__Lachnospirales;f__Lachnospiraceae;g__Blautia_A;s__[Blautia_A_Flurry_p2_metabat
PDL1vs.k__Bacteria;p__Firmicutes_A;c__Clostridia;o__Lachnospirales;f__Lachnospiraceae;g__Blautia_A;s__[Blautia_A_Garnier_p1_metaba
PDL1vs.k__Bacteria;p__Firmicutes_A;c__Clostridia;o__Lachnospirales;f__Lachnospiraceae;g__Blautia_A;s__[Blautia_A_Glacier_p2_metaba
PDL1vs.k__Bacteria;p__Firmicutes_A;c__Clostridia;o__Lachnospirales;f__Lachnospiraceae;g__Blautia_A;s__[Blautia_A_Oklahoma_p2_max
PDL1vs.k__Bacteria;p__Firmicutes_A;c__Clostridia;o__Lachnospirales;f__Lachnospiraceae;g__Blautia_A;s__[Blautia_A_Pinky_p2_maxbin2_
PDL1vs.k__Bacteria;p__Firmicutes_A;c__Clostridia;o__Lachnospirales;f__Lachnospiraceae;g__Blautia_A;s__[Blautia_A_Scooby_p2_maxbin
PDL1vs.k__Bacteria;p__Firmicutes_A;c__Clostridia;o__Lachnospirales;f__Lachnospiraceae;g__Blautia_A;s__[Blautia_A_Skinner_p2_maxbin
PDL1vs.k__Bacteria;p__Firmicutes_A;c__Clostridia;o__Lachnospirales;f__Lachnospiraceae;g__Blautia_A;s__[Blautia_A_Stewie_p2_metabat
PDL1vs.k__Bacteria;p__Firmicutes_A;c__Clostridia;o__Lachnospirales;f__Lachnospiraceae;g__Blautia_A;s__[Blautia_A_Virginia_p1_maxbin
PDL1vs.k__Bacteria;p__Firmicutes_A;c__Clostridia;o__Lachnospirales;f__Lachnospiraceae;g__CAG-81;s__CAG-81_sp000435795
PDL1vs.k__Bacteria;p__Firmicutes_A;c__Clostridia;o__Lachnospirales;f__Lachnospiraceae;g__CAG-81;s__Other
PDL1vs.k__Bacteria;p__Firmicutes_A;c__Clostridia;o__Lachnospirales;f__Lachnospiraceae;g__Clostridium_Q;s__Clostridium_Q_sp000435
PDL1vs.k__Bacteria;p__Firmicutes_A;c__Clostridia;o__Lachnospirales;f__Lachnospiraceae;g__Clostridium_Q;s__Clostridium_Q_sp003024

PDL1vs.k__Bacteria;p__Firmicutes_A;c__Clostridia;o__Lachnospirales;f__Lachnospiraceae;g__Clostridium_Q;s__Other
PDL1vs.k__Bacteria;p__Firmicutes_A;c__Clostridia;o__Lachnospirales;f__Lachnospiraceae;g__Clostridium_Q;s__[Clostridium_Q_Kit_p2_m
PDL1vs.k__Bacteria;p__Firmicutes_A;c__Clostridia;o__Lachnospirales;f__Lachnospiraceae;g__Dorea;s__Dorea_formicigenerans
PDL1vs.k__Bacteria;p__Firmicutes_A;c__Clostridia;o__Lachnospirales;f__Lachnospiraceae;g__Dorea_A;s__Dorea_A_longicatena
PDL1vs.k__Bacteria;p__Firmicutes_A;c__Clostridia;o__Lachnospirales;f__Lachnospiraceae;g__Dorea_A;s__Dorea_A_longicatena_B
PDL1vs.k__Bacteria;p__Firmicutes_A;c__Clostridia;o__Lachnospirales;f__Lachnospiraceae;g__Dorea_B;s__Dorea_B_phocaeensis
PDL1vs.k__Bacteria;p__Firmicutes_A;c__Clostridia;o__Lachnospirales;f__Lachnospiraceae;g__Eisenbergiella;s__Eisenbergiella_sp9005397
PDL1vs.k__Bacteria;p__Firmicutes_A;c__Clostridia;o__Lachnospirales;f__Lachnospiraceae;g__Enterocloster;s__Enterocloster_bolteae
PDL1vs.k__Bacteria;p__Firmicutes_A;c__Clostridia;o__Lachnospirales;f__Lachnospiraceae;g__Enterocloster;s__Enterocloster_clostridiofor
PDL1vs.k__Bacteria;p__Firmicutes_A;c__Clostridia;o__Lachnospirales;f__Lachnospiraceae;g__Enterocloster;s__Enterocloster_sp00043137
PDL1vs.k__Bacteria;p__Firmicutes_A;c__Clostridia;o__Lachnospirales;f__Lachnospiraceae;g__Enterocloster;s__Enterocloster_sp00151762
PDL1vs.k__Bacteria;p__Firmicutes_A;c__Clostridia;o__Lachnospirales;f__Lachnospiraceae;g__Enterocloster;s__Enterocloster_sp90053848
PDL1vs.k__Bacteria;p__Firmicutes_A;c__Clostridia;o__Lachnospirales;f__Lachnospiraceae;g__Enterocloster;s__Other
PDL1vs.k__Bacteria;p__Firmicutes_A;c__Clostridia;o__Lachnospirales;f__Lachnospiraceae;g__Eubacterium_I;s__Eubacterium_I_ramulus
PDL1vs.k__Bacteria;p__Firmicutes_A;c__Clostridia;o__Lachnospirales;f__Lachnospiraceae;g__Faecalimonas;s__Faecalimonas_sp9005502
PDL1vs.k__Bacteria;p__Firmicutes_A;c__Clostridia;o__Lachnospirales;f__Lachnospiraceae;g__Faecalimonas;s__Faecalimonas_sp9005518
PDL1vs.k__Bacteria;p__Firmicutes_A;c__Clostridia;o__Lachnospirales;f__Lachnospiraceae;g__Faecalimonas;s__Faecalimonas_umbilicata
PDL1vs.k__Bacteria;p__Firmicutes_A;c__Clostridia;o__Lachnospirales;f__Lachnospiraceae;g__Faecalimonas;s__Other
PDL1vs.k__Bacteria;p__Firmicutes_A;c__Clostridia;o__Lachnospirales;f__Lachnospiraceae;g__Faecalimonas;s__[Faecalimonas_Bissell_p2
PDL1vs.k__Bacteria;p__Firmicutes_A;c__Clostridia;o__Lachnospirales;f__Lachnospiraceae;g__Faecalimonas;s__[Faecalimonas_Flurry_p2_
PDL1vs.k__Bacteria;p__Firmicutes_A;c__Clostridia;o__Lachnospirales;f__Lachnospiraceae;g__Faecalimonas;s__[Faecalimonas_Skinner_p
PDL1vs.k__Bacteria;p__Firmicutes_A;c__Clostridia;o__Lachnospirales;f__Lachnospiraceae;g__Fusicatenibacter;s__Fusicatenibacter_sacch
PDL1vs.k__Bacteria;p__Firmicutes_A;c__Clostridia;o__Lachnospirales;f__Lachnospiraceae;g__Mediterraneibacter;s__Mediterraneibacter_fa
PDL1vs.k__Bacteria;p__Firmicutes_A;c__Clostridia;o__Lachnospirales;f__Lachnospiraceae;g__Mediterraneibacter;s__Mediterraneibacter_la
PDL1vs.k__Bacteria;p__Firmicutes_A;c__Clostridia;o__Lachnospirales;f__Lachnospiraceae;g__Mediterraneibacter;s__Mediterraneibacter_m
PDL1vs.k__Bacteria;p__Firmicutes_A;c__Clostridia;o__Lachnospirales;f__Lachnospiraceae;g__Mediterraneibacter;s__Mediterraneibacter_t
PDL1vs.k__Bacteria;p__Firmicutes_A;c__Clostridia;o__Lachnospirales;f__Lachnospiraceae;g__Other;s__Other
PDL1vs.k__Bacteria;p__Firmicutes_A;c__Clostridia;o__Lachnospirales;f__Lachnospiraceae;g__Roseburia;s__Roseburia_sp900548205
PDL1vs.k__Bacteria;p__Firmicutes_A;c__Clostridia;o__Lachnospirales;f__Lachnospiraceae;g__Roseburia;s__[Roseburia_Garnier_p1_meta
PDL1vs.k__Bacteria;p__Firmicutes_A;c__Clostridia;o__Lachnospirales;f__Lachnospiraceae;g__Ruminococcus_A;s__Ruminococcus_A_sp0
PDL1vs.k__Bacteria;p__Firmicutes_A;c__Clostridia;o__Lachnospirales;f__Lachnospiraceae;g__Ruminococcus_B;s__Other

PDL1vs.k__Bacteria;p__Firmicutes_A;c__Clostridia;o__Lachnospirales;f__Lachnospiraceae;g__Ruminococcus_B;s__Ruminococcus_B_gna
PDL1vs.k__Bacteria;p__Firmicutes_A;c__Clostridia;o__Lachnospirales;f__Lachnospiraceae;g__Ruminococcus_B;s__[Ruminococcus_B_Sn
PDL1vs.k__Bacteria;p__Firmicutes_A;c__Clostridia;o__Lachnospirales;f__Lachnospiraceae;g__Schaedlerella;s__Schaedlerella_glycyrrhizini
PDL1vs.k__Bacteria;p__Firmicutes_A;c__Clostridia;o__Lachnospirales;f__Lachnospiraceae;g__Schaedlerella;s__Schaedlerella_sp90076597
PDL1vs.k__Bacteria;p__Firmicutes_A;c__Clostridia;o__Lachnospirales;f__Lachnospiraceae;g__Schaedlerella;s__[Schaedlerella_Snoopy_p2
PDL1vs.k__Bacteria;p__Firmicutes_A;c__Clostridia;o__Lachnospirales;f__Lachnospiraceae;g__UBA9502;s__UBA9502_sp003481825
PDL1vs.k__Bacteria;p__Firmicutes_A;c__Clostridia;o__Lachnospirales;f__Lachnospiraceae;g__UBA9502;s__UBA9502_sp900538475
PDL1vs.k__Bacteria;p__Firmicutes_A;c__Clostridia;o__Oscillospirales;f__Butyricicoccaceae;g__Agathobaculum;s__Agathobaculum_sp9002
PDL1vs.k__Bacteria;p__Firmicutes_A;c__Clostridia;o__Oscillospirales;f__Butyricicoccaceae;g__Butyricicoccus;s__Butyricicoccus_pullicae
PDL1vs.k__Bacteria;p__Firmicutes_A;c__Clostridia;o__Oscillospirales;f__Oscillospiraceae;g__CAG-110;s__[CAG-110_Escalade_p2_maxbin2
PDL1vs.k__Bacteria;p__Firmicutes_A;c__Clostridia;o__Oscillospirales;f__Ruminococcaceae;g__Faecalibacterium;s__Faecalibacterium_sp9
PDL1vs.k__Bacteria;p__Firmicutes_A;c__Clostridia;o__Oscillospirales;f__Ruminococcaceae;g__Faecalibacterium;s__Other
PDL1vs.k__Bacteria;p__Firmicutes_A;c__Clostridia;o__Oscillospirales;f__Ruminococcaceae;g__Fournierella;s__Fournierella_massiliensis
PDL1vs.k__Bacteria;p__Firmicutes_A;c__Clostridia;o__Oscillospirales;f__Ruminococcaceae;g__Fournierella;s__[Fournierella_Flurry_p2_ma
PDL1vs.k__Bacteria;p__Firmicutes_A;c__Clostridia;o__Oscillospirales;f__Ruminococcaceae;g__Negativibacillus;s__Negativibacillus_sp000
PDL1vs.k__Bacteria;p__Firmicutes_A;c__Clostridia;o__Oscillospirales;f__Ruminococcaceae;g__UMGS966;s__Other
PDL1vs.k__Bacteria;p__Firmicutes_A;c__Clostridia;o__Oscillospirales;f__Ruminococcaceae;g__UMGS966;s__[UMGS966_Kit_p2_maxbin2_l
PDL1vs.k__Bacteria;p__Firmicutes_A;c__Clostridia;o__Oscillospirales;f__Ruminococcaceae;g__UMGS966;s__[UMGS966_Oklahoma_p2_me
PDL1vs.k__Bacteria;p__Firmicutes_A;c__Clostridia;o__Oscillospirales;f__Ruminococcaceae;g__UMGS966;s__[UMGS966_Peterbilt_p2_maxb
PDL1vs.k__Bacteria;p__Firmicutes_A;c__Clostridia;o__Other;f__Other;g__Other;s__Other
PDL1vs.k__Bacteria;p__Firmicutes_A;c__Clostridia;o__Peptostreptococcales;f__Other;g__Other;s__Other
PDL1vs.k__Bacteria;p__Firmicutes_A;c__Clostridia;o__Peptostreptococcales;f__Peptostreptococcaceae;g__Clostridioides;s__Clostridioides
PDL1vs.k__Bacteria;p__Firmicutes_A;c__Clostridia;o__Peptostreptococcales;f__Peptostreptococcaceae;g__Other;s__Other
PDL1vs.k__Bacteria;p__Firmicutes_A;c__Clostridia;o__Peptostreptococcales;f__Peptostreptococcaceae;g__Paraclostridium;s__Other
PDL1vs.k__Bacteria;p__Firmicutes_A;c__Clostridia;o__Peptostreptococcales;f__Peptostreptococcaceae;g__Paraclostridium;s__Paraclostric
PDL1vs.k__Bacteria;p__Firmicutes_A;c__Clostridia;o__Peptostreptococcales;f__Peptostreptococcaceae;g__Peptacetobacter;s__Other
PDL1vs.k__Bacteria;p__Firmicutes_A;c__Clostridia;o__Peptostreptococcales;f__Peptostreptococcaceae;g__Peptacetobacter;s__Peptacetob
PDL1vs.k__Bacteria;p__Firmicutes_A;c__Clostridia;o__Peptostreptococcales;f__Peptostreptococcaceae;g__Peptacetobacter;s__Peptacetob
PDL1vs.k__Bacteria;p__Firmicutes_A;c__Clostridia;o__Peptostreptococcales;f__Peptostreptococcaceae;g__Peptacetobacter;s__[Peptacetol
PDL1vs.k__Bacteria;p__Firmicutes_A;c__Clostridia;o__Peptostreptococcales;f__Peptostreptococcaceae;g__Peptacetobacter;s__[Peptacetol
PDL1vs.k__Bacteria;p__Firmicutes_A;c__Clostridia;o__Peptostreptococcales;f__Peptostreptococcaceae;g__Romboutsia;s__Other

PDL1vs.k__Bacteria;p__Firmicutes_A;c__Clostridia;o__Peptostreptococcales;f__Peptostreptococcaceae;g__Romboutsia;s__Romboutsia_hc
PDL1vs.k__Bacteria;p__Firmicutes_A;c__Clostridia;o__Peptostreptococcales;f__Peptostreptococcaceae;g__Romboutsia;s__Romboutsia_ile
PDL1vs.k__Bacteria;p__Firmicutes_A;c__Clostridia;o__Peptostreptococcales;f__Peptostreptococcaceae;g__Romboutsia;s__[Romboutsia_B
PDL1vs.k__Bacteria;p__Firmicutes_A;c__Clostridia;o__Peptostreptococcales;f__Peptostreptococcaceae;g__Terrisporobacter;s__Terrisporob
PDL1vs.k__Bacteria;p__Firmicutes_B;c__Peptococcia;o__Peptococcales;f__Peptococcaceae;g__UMGS1590;s__UMGS1590_sp900553245
PDL1vs.k__Bacteria;p__Firmicutes_C;c__Negativicutes;o__Acidaminococcales;f__Acidaminococcaceae;g__Phascolarctobacterium_A;s__Ph
PDL1vs.k__Bacteria;p__Firmicutes_C;c__Negativicutes;o__Selenomonadales;f__Selenomonadaceae;g__Megamonas;s__Megamonas_funifo
PDL1vs.k__Bacteria;p__Firmicutes_C;c__Negativicutes;o__Selenomonadales;f__Selenomonadaceae;g__Megamonas;s__Other
PDL1vs.k__Bacteria;p__Firmicutes_C;c__Negativicutes;o__Selenomonadales;f__Selenomonadaceae;g__Megamonas;s__[Megamonas_Belle
PDL1vs.k__Bacteria;p__Fusobacteriota;c__Fusobacteriia;o__Fusobacteriales;f__Fusobacteriaceae;g__Fusobacterium_A;s__Fusobacterium_
PDL1vs.k__Bacteria;p__Fusobacteriota;c__Fusobacteriia;o__Fusobacteriales;f__Fusobacteriaceae;g__Fusobacterium_A;s__Fusobacterium_
PDL1vs.k__Bacteria;p__Fusobacteriota;c__Fusobacteriia;o__Fusobacteriales;f__Fusobacteriaceae;g__Fusobacterium_A;s__Other
PDL1vs.k__Bacteria;p__Fusobacteriota;c__Fusobacteriia;o__Fusobacteriales;f__Fusobacteriaceae;g__Fusobacterium_B;s__Fusobacterium_
PDL1vs.k__Bacteria;p__Fusobacteriota;c__Fusobacteriia;o__Fusobacteriales;f__Fusobacteriaceae;g__Fusobacterium_B;s__Fusobacterium_
PDL1vs.k__Bacteria;p__Fusobacteriota;c__Fusobacteriia;o__Fusobacteriales;f__Fusobacteriaceae;g__Fusobacterium_B;s__Other
PDL1vs.k__Bacteria;p__Fusobacteriota;c__Fusobacteriia;o__Fusobacteriales;f__Fusobacteriaceae;g__Other;s__Other
PDL1vs.k__Bacteria;p__Other;c__Other;o__Other;f__Other;g__Other;s__Other
PDL1vs.k__Bacteria;p__Proteobacteria;c__Gammaproteobacteria;o__Burkholderiales;f__Burkholderiaceae;g__Sutterella;s__Other
PDL1vs.k__Bacteria;p__Proteobacteria;c__Gammaproteobacteria;o__Enterobacterales;f__Enterobacteriaceae;g__Escherichia;s__Escherichi
PDL1vs.k__Bacteria;p__Proteobacteria;c__Gammaproteobacteria;o__Enterobacterales;f__Enterobacteriaceae;g__Escherichia;s__Other
PDL1vs.k__Bacteria;p__Proteobacteria;c__Gammaproteobacteria;o__Enterobacterales;f__Enterobacteriaceae;g__Other;s__Other

Spearman r	95% confidence interval	P (two-tailed)	P value summary	Exact or approximate P value?
-0.333	-0.7693 to 0.3156	0.2888	ns	Exact
0.414	-0.2281 to 0.8052	0.1803	ns	Exact
0.167	-0.4652 to 0.6865	0.6007	ns	Exact
0.336	-0.3120 to 0.7709	0.283	ns	Exact
-0.336	-0.7709 to 0.3120	0.283	ns	Exact
0.013	-0.5780 to 0.5952	0.9692	ns	Exact
0.417	-0.2249 to 0.8063	0.178	ns	Exact
0.532	-0.07898 to 0.8527	0.0776	ns	Exact
0.161	-0.4700 to 0.6832	0.6146	ns	Exact
0.617	0.04727 to 0.8837	0.0358	*	Exact
-0.228	-0.7187 to 0.4139	0.4704	ns	Exact
-0.333	-0.7693 to 0.3156	0.2888	ns	Exact
0.414	-0.2281 to 0.8052	0.1803	ns	Exact
0.1671	-0.4652 to 0.6865	0.6007	ns	Exact
0.3363	-0.3120 to 0.7709	0.283	ns	Exact
-0.3363	-0.7709 to 0.3120	0.283	ns	Exact
0.01311	-0.5780 to 0.5952	0.9692	ns	Exact
0.4168	-0.2249 to 0.8063	0.178	ns	Exact
0.5324	-0.07898 to 0.8527	0.0776	ns	Exact
0.1611	-0.4700 to 0.6832	0.6146	ns	Exact
0.6169	0.04727 to 0.8837	0.0358	*	Exact
-0.1943	-0.7011 to 0.4429	0.5424	ns	Exact
-0.3556	-0.7796 to 0.2920	0.2542	ns	Exact
-0.3328	-0.7693 to 0.3156	0.2888	ns	Exact
0.414	-0.2281 to 0.8052	0.1803	ns	Exact
0.1671	-0.4652 to 0.6865	0.6007	ns	Exact
0.1576	-0.4728 to 0.6813	0.6231	ns	Exact
0.2129	-0.4272 to 0.7108	0.4995	ns	Exact
0.2837	-0.3635 to 0.7462	0.369	ns	Exact
0.3499	-0.2980 to 0.7771	0.2619	ns	Exact
-0.2767	-0.7428 to 0.3701	0.3814	ns	Exact
-0.3398	-0.7725 to 0.3084	0.2783	ns	Exact

0.2439	-0.4001 to 0.7266	0.4395	ns	Exact
-0.2067	-0.7076 to 0.4325	0.5168	ns	Exact
-0.1506	-0.6775 to 0.4784	0.6389	ns	Exact
0.01311	-0.5780 to 0.5952	0.9692	ns	Exact
0.5891	0.003611 to 0.8738	0.0472	*	Exact
0.2035	-0.4352 to 0.7059	0.5218	ns	Exact
0.5324	-0.07898 to 0.8527	0.0776	ns	Exact
0.1611	-0.4700 to 0.6832	0.6146	ns	Exact
0.353	-0.2948 to 0.7785	0.2568	ns	Exact
0.5598	-0.04009 to 0.8630	0.0615	ns	Exact
-0.1397	-0.6714 to 0.4869	0.6637	ns	Exact
-0.3556	-0.7796 to 0.2920	0.2542	ns	Exact
-0.1185	-0.6594 to 0.5032	0.7237	ns	Exact
-0.3328	-0.7693 to 0.3156	0.2888	ns	Exact
0.6561	0.1127 to 0.8974	0.024	*	Exact
0.1312	-0.4935 to 0.6666	0.6908	ns	Exact
0.4028	-0.2408 to 0.8004	0.1941	ns	Exact
0.3203	-0.3280 to 0.7635	0.3051	ns	Exact
0.5448	-0.06162 to 0.8574	0.0702	ns	Exact
0.09457	-0.5211 to 0.6455	0.7704	ns	Exact
0.3573	-0.2903 to 0.7804	0.2526	ns	Exact
0.3874	-0.2580 to 0.7937	0.2227	ns	Exact
0.2129	-0.4272 to 0.7108	0.4995	ns	Exact
0.4334	-0.2055 to 0.8133	0.1601	ns	Exact
0.09507	-0.5207 to 0.6458	0.7665	ns	Exact
0.2332	-0.4095 to 0.7212	0.4613	ns	Exact
0.3499	-0.2980 to 0.7771	0.2619	ns	Exact
-0.2767	-0.7428 to 0.3701	0.3814	ns	Exact
-0.3398	-0.7725 to 0.3084	0.2783	ns	Exact
0.6477	0.09837 to 0.8945	0.0266	*	Exact
0.4582	-0.1758 to 0.8235	0.1345	ns	Exact

0.1331	-0.4920 to 0.6677	0.6785	ns	Exact
-0.2067	-0.7076 to 0.4325	0.5168	ns	Exact
-0.3126	-0.7600 to 0.3356	0.3187	ns	Exact
-0.1506	-0.6775 to 0.4784	0.6389	ns	Exact
0.01311	-0.5780 to 0.5952	0.9692	ns	Exact
0.5891	0.003611 to 0.8738	0.0472	*	Exact
0.2035	-0.4352 to 0.7059	0.5218	ns	Exact
0.5324	-0.07898 to 0.8527	0.0776	ns	Exact
0.1611	-0.4700 to 0.6832	0.6146	ns	Exact
0.353	-0.2948 to 0.7785	0.2568	ns	Exact
0.6382	0.08236 to 0.8912	0.0292	*	Exact
0.09655	-0.5196 to 0.6466	0.7659	ns	Exact
0.113	-0.5074 to 0.6562	0.7364	ns	Exact
-0.3556	-0.7796 to 0.2920	0.2542	ns	Exact
-0.3328	-0.7693 to 0.3156	0.2888	ns	Exact
-0.2104	-0.7095 to 0.4293	0.5066	ns	Exact
0.6061	0.03015 to 0.8799	0.0404	*	Exact
0.1312	-0.4935 to 0.6666	0.6908	ns	Exact
0.5725	-0.02147 to 0.8677	0.0551	ns	Exact
0.4359	-0.2027 to 0.8143	0.1564	ns	Exact
0.1793	-0.4553 to 0.6931	0.5826	ns	Exact
0.5219	-0.09343 to 0.8487	0.0847	ns	Exact
0.2845	-0.3628 to 0.7466	0.3647	ns	Exact
0.1702	-0.4627 to 0.6882	0.592	ns	Exact
0.3203	-0.3280 to 0.7635	0.3051	ns	Exact
0.5448	-0.06162 to 0.8574	0.0702	ns	Exact
0.2544	-0.3906 to 0.7319	0.4202	ns	Exact
-0.02116	-0.6004 to 0.5727	0.9515	ns	Exact
0.5425	-0.06484 to 0.8566	0.0727	ns	Exact
-0.3657	-0.7842 to 0.2814	0.2388	ns	Exact
-0.3671	-0.7848 to 0.2799	0.2369	ns	Exact

-0.3683	-0.7853 to 0.2786	0.2356	ns	Exact
0.3298	-0.3185 to 0.7679	0.2908	ns	Exact
-0.06714	-0.6291 to 0.5409	0.8356	ns	Exact
-0.01767	-0.5982 to 0.5750	0.9588	ns	Exact
0.5871	0.0005886 to 0.8731	0.048	*	Exact
0.4468	-0.1896 to 0.8189	0.1454	ns	Exact
0.4121	-0.2303 to 0.8043	0.1817	ns	Exact
0.08451	-0.5284 to 0.6395	0.792	ns	Exact
0.3874	-0.2580 to 0.7937	0.2227	ns	Exact
0.2129	-0.4272 to 0.7108	0.4995	ns	Exact
-0.03581	-0.6097 to 0.5627	0.9128	ns	Exact
0.1188	-0.5030 to 0.6595	0.7109	ns	Exact
0.2251	-0.4167 to 0.7171	0.4747	ns	Exact
0.2529	-0.3920 to 0.7311	0.4288	ns	Exact
0.3639	-0.2833 to 0.7833	0.2421	ns	Exact
0.1378	-0.4884 to 0.6703	0.6662	ns	Exact
0.3499	-0.2980 to 0.7771	0.2619	ns	Exact
-0.0246	-0.6026 to 0.5703	0.9406	ns	Exact
-0.261	-0.7352 to 0.3846	0.409	ns	Exact
-0.133	-0.6676 to 0.4921	0.676	ns	Exact
-0.4565	-0.8228 to 0.1778	0.1409	ns	Exact
-0.02507	-0.6029 to 0.5700	0.9377	ns	Exact
0.2299	-0.4124 to 0.7196	0.4722	ns	Exact
-0.5359	-0.8541 to 0.07411	0.0756	ns	Exact
-0.03152	-0.6070 to 0.5656	0.9258	ns	Exact
0.2496	-0.3950 to 0.7295	0.4287	ns	Exact
0.3573	-0.2903 to 0.7804	0.2526	ns	Exact
-0.1169	-0.6585 to 0.5044	0.7155	ns	Exact
0.6042	0.02707 to 0.8792	0.0422	*	Exact
-0.1428	-0.6731 to 0.4845	0.6535	ns	Exact

0.1908	-0.4459 to 0.6992	0.5477	ns	Exact
0.005272	-0.5832 to 0.5902	0.9889	ns	Exact
-0.09107	-0.6434 to 0.5236	0.7782	ns	Exact
0.04286	-0.5579 to 0.6141	0.8944	ns	Exact
0.4168	-0.2249 to 0.8063	0.178	ns	Exact
0.2568	-0.3884 to 0.7331	0.4187	ns	Exact
-0.2857	-0.7472 to 0.3616	0.3634	ns	Exact
-0.6673	-0.9012 to -0.1324	0.0205	*	Exact
-0.3211	-0.7639 to 0.3273	0.3048	ns	Exact
0.2929	-0.3548 to 0.7506	0.3508	ns	Exact
-0.3743	-0.7880 to 0.2721	0.2282	ns	Exact
-0.4273	-0.8108 to 0.2127	0.1662	ns	Exact
-0.3783	-0.7897 to 0.2679	0.2242	ns	Exact
0.1476	-0.4807 to 0.6758	0.6432	ns	Exact
0.261	-0.3846 to 0.7351	0.4121	ns	Exact
0.7268	0.2442 to 0.9208	0.0102	*	Exact
0.4939	-0.1307 to 0.8378	0.1045	ns	Exact
0.01511	-0.5767 to 0.5965	0.9665	ns	Exact
0.1529	-0.4766 to 0.6787	0.6314	ns	Exact
0.3915	-0.2534 to 0.7955	0.2057	ns	Exact
-0.3251	-0.7657 to 0.3233	0.2997	ns	Exact
0.2561	-0.3890 to 0.7327	0.4176	ns	Exact
-0.2067	-0.7076 to 0.4325	0.5168	ns	Exact
-0.3126	-0.7600 to 0.3356	0.3187	ns	Exact
-0.05923	-0.6243 to 0.5465	0.8694	ns	Exact
-0.2403	-0.7248 to 0.4033	0.448	ns	Exact
-0.1473	-0.6756 to 0.4810	0.6434	ns	Exact
-0.1751	-0.6909 to 0.4587	0.5835	ns	Exact
-0.1683	-0.6872 to 0.4642	0.5965	ns	Exact
0.1716	-0.4616 to 0.6890	0.592	ns	Exact
-0.05923	-0.6243 to 0.5465	0.8409	ns	Exact

0.01311	-0.5780 to 0.5952	0.9692	ns	Exact
0.318	-0.3303 to 0.7625	0.3105	ns	Exact
0.2035	-0.4352 to 0.7059	0.5218	ns	Exact
0.4623	-0.1707 to 0.8252	0.1314	ns	Exact
0.4576	-0.1765 to 0.8233	0.1346	ns	Exact
0.1403	-0.4864 to 0.6717	0.6591	ns	Exact
0.1611	-0.4700 to 0.6832	0.6146	ns	Exact
0.3858	-0.2597 to 0.7930	0.2129	ns	Exact
0.6382	0.08236 to 0.8912	0.0292	*	Exact
0.6414	0.08768 to 0.8923	0.0282	*	Exact
-0.3608	-0.7820 to 0.2866	0.2481	ns	Exact
-0.4542	-0.8219 to 0.1806	0.1382	ns	Exact
-0.3579	-0.7807 to 0.2897	0.2501	ns	Exact
-0.6672	-0.9012 to -0.1323	0.0212	*	Exact
-0.1807	-0.6939 to 0.4542	0.569	ns	Exact
-0.2561	-0.7327 to 0.3890	0.4178	ns	Exact
-0.2104	-0.7095 to 0.4293	0.5066	ns	Exact
0.5924	0.008749 to 0.8750	0.0458	*	Exact
0.207	-0.4322 to 0.7078	0.5136	ns	Exact
0.1312	-0.4935 to 0.6666	0.6908	ns	Exact
0.08762	-0.5261 to 0.6414	0.7909	ns	Exact
0.2636	-0.3822 to 0.7364	0.4029	ns	Exact
0.5738	-0.01940 to 0.8682	0.0548	ns	Exact
0.4359	-0.2027 to 0.8143	0.1564	ns	Exact
0.7569	0.3061 to 0.9304	0.0064	**	Exact
0.4062	-0.2370 to 0.8018	0.1908	ns	Exact
0.4428	-0.1944 to 0.8172	0.1491	ns	Exact
0.1611	-0.4700 to 0.6832	0.6146	ns	Exact
0.1253	-0.4981 to 0.6632	0.6941	ns	Exact
0.2105	-0.4292 to 0.7096	0.5079	ns	Exact
0.2845	-0.3628 to 0.7466	0.3647	ns	Exact

0.1592	-0.4716 to 0.6822	0.6158	ns	Exact
0.1235	-0.4994 to 0.6622	0.7015	ns	Exact
0.3072	-0.3410 to 0.7574	0.3263	ns	Exact
0.5448	-0.06162 to 0.8574	0.0702	ns	Exact
0.2544	-0.3906 to 0.7319	0.4202	ns	Exact
-0.03719	-0.6106 to 0.5618	0.9081	ns	Exact
-0.05418	-0.6211 to 0.5500	0.8675	ns	Exact
0.1421	-0.4851 to 0.6727	0.6613	ns	Exact
-0.03926	-0.6119 to 0.5604	0.9037	ns	Exact
0.4146	-0.2274 to 0.8054	0.1894	ns	Exact
-0.3657	-0.7842 to 0.2814	0.2388	ns	Exact
-0.3671	-0.7848 to 0.2799	0.2369	ns	Exact
-0.3683	-0.7853 to 0.2786	0.2356	ns	Exact
0.3298	-0.3185 to 0.7679	0.2908	ns	Exact
-0.06714	-0.6291 to 0.5409	0.8356	ns	Exact
-0.01767	-0.5982 to 0.5750	0.9588	ns	Exact
0.5871	0.0005886 to 0.8731	0.048	*	Exact
0.5195	-0.09671 to 0.8478	0.0863	ns	Exact
0.4121	-0.2303 to 0.8043	0.1817	ns	Exact
0.08451	-0.5284 to 0.6395	0.792	ns	Exact
0.3874	-0.2580 to 0.7937	0.2227	ns	Exact
0.1797	-0.4550 to 0.6933	0.5746	ns	Exact
0.3783	-0.2679 to 0.7897	0.2249	ns	Exact
0.2529	-0.3920 to 0.7311	0.4288	ns	Exact
0.2529	-0.3920 to 0.7311	0.4288	ns	Exact
0.2248	-0.4169 to 0.7169	0.4765	ns	Exact
0.3499	-0.2980 to 0.7771	0.2619	ns	Exact
0.3905	-0.2545 to 0.7950	0.208	ns	Exact
-0.09877	-0.6479 to 0.5180	0.7598	ns	Exact
-0.133	-0.6676 to 0.4921	0.676	ns	Exact
-0.4565	-0.8228 to 0.1778	0.1409	ns	Exact

0.2299	-0.4124 to 0.7196	0.4722	ns	Exact
-0.4812	-0.8328 to 0.1470	0.1667	ns	Exact
-0.4063	-0.8019 to 0.2369	0.1896	ns	Exact
-0.4812	-0.8328 to 0.1470	0.1667	ns	Exact
-0.2596	-0.7345 to 0.3858	0.4112	ns	Exact
-0.09825	-0.6476 to 0.5183	0.76	ns	Exact
-0.3678	-0.7851 to 0.2792	0.2385	ns	Exact
-0.1376	-0.6702 to 0.4886	0.6655	ns	Exact
-0.4553	-0.8224 to 0.1793	0.138	ns	Exact
0.03152	-0.5656 to 0.6070	0.9258	ns	Exact
-0.03221	-0.6074 to 0.5652	0.9216	ns	Exact
-0.2257	-0.7174 to 0.4161	0.4855	ns	Exact
-0.429	-0.8115 to 0.2108	0.1637	ns	Exact
0.04905	-0.5536 to 0.6180	0.9078	ns	Exact
-0.2662	-0.7377 to 0.3798	0.3998	ns	Exact
0.4102	-0.2325 to 0.8035	0.184	ns	Exact
0.04375	-0.5573 to 0.6147	>0.9999	ns	Exact
0.1716	-0.4616 to 0.6890	0.592	ns	Exact
0.1734	-0.4601 to 0.6899	0.5881	ns	Exact
0.07938	-0.5321 to 0.6365	0.8072	ns	Exact
0.04178	-0.5586 to 0.6135	0.8985	ns	Exact
0.1526	-0.4768 to 0.6786	0.6314	ns	Exact
-0.1954	-0.7017 to 0.4420	0.5375	ns	Exact
-0.02507	-0.6029 to 0.5700	0.9377	ns	Exact
0.2596	-0.3859 to 0.7344	0.4545	ns	Exact
0.03632	-0.5624 to 0.6100	0.914	ns	Exact
0.07046	-0.5385 to 0.6311	0.8276	ns	Exact
0.165	-0.4669 to 0.6854	0.6041	ns	Exact
0.3437	-0.3044 to 0.7743	0.2725	ns	Exact
0.1775	-0.4568 to 0.6921	0.5764	ns	Exact
-0.3508	-0.7775 to 0.2971	0.2587	ns	Exact

0.2558	-0.3894 to 0.7326	0.4394	ns	Exact
0.4383	-0.1998 to 0.8154	0.1536	ns	Exact
0.6042	0.02707 to 0.8792	0.0422	*	Exact
-0.2179	-0.7134 to 0.4228	0.4893	ns	Exact
-0.1003	-0.6488 to 0.5168	0.7598	ns	Exact
0.1908	-0.4459 to 0.6992	0.5477	ns	Exact
0.005272	-0.5832 to 0.5902	0.9889	ns	Exact
0.1752	-0.4586 to 0.6909	0.5833	ns	Exact
-0.5073	-0.8431 to 0.1131	0.097	ns	Exact
0.1451	-0.4827 to 0.6744	0.6688	ns	Exact
-0.09107	-0.6434 to 0.5236	0.7782	ns	Exact
0.3635	-0.2837 to 0.7832	0.2433	ns	Exact
0.04286	-0.5579 to 0.6141	0.8944	ns	Exact
-0.1053	-0.6517 to 0.5131	0.7429	ns	Exact
-0.1681	-0.6871 to 0.4644	0.5989	ns	Exact
-0.1681	-0.6871 to 0.4644	0.5989	ns	Exact
0.1541	-0.4756 to 0.6794	0.6302	ns	Exact
0.3561	-0.2915 to 0.7799	0.2526	ns	Exact
-0.121	-0.6608 to 0.5013	0.7054	ns	Exact
-0.01754	-0.5981 to 0.5751	0.9591	ns	Exact
0.2568	-0.3884 to 0.7331	0.4187	ns	Exact
-0.5668	-0.8657 to 0.02978	0.0572	ns	Exact
0.4542	-0.1806 to 0.8219	0.1667	ns	Exact
-0.5257	-0.8502 to 0.08821	0.0818	ns	Exact
-0.4381	-0.8153 to 0.2000	0.1551	ns	Exact
-0.3211	-0.7639 to 0.3273	0.3048	ns	Exact
0.07577	-0.5347 to 0.6343	0.8146	ns	Exact
0.07521	-0.5351 to 0.6339	0.8239	ns	Exact
-0.3743	-0.7880 to 0.2721	0.2282	ns	Exact
-0.4042	-0.8010 to 0.2392	0.1912	ns	Exact

-0.2452	-0.7273 to 0.3989	0.4392	ns	Exact
-0.3915	-0.7955 to 0.2534	0.2072	ns	Exact
0.1576	-0.4728 to 0.6813	0.6231	ns	Exact
-0.3316	-0.7687 to 0.3168	0.2898	ns	Exact
-0.5954	-0.8761 to -0.01330	0.0454	*	Exact
0	-0.5867 to 0.5867	>0.9999	ns	Exact
0.2011	-0.4372 to 0.7046	0.5274	ns	Exact
**:91975**				
0.7268	0.2442 to 0.9208	0.0102	*	Exact
0.561	-0.03835 to 0.8635	0.0613	ns	Exact
0.1529	-0.4766 to 0.6787	0.6314	ns	Exact
0.2667	-0.3793 to 0.7380	0.4	ns	Exact
0.4041	-0.2394 to 0.8009	0.1916	ns	Exact
0.2299	-0.4125 to 0.7195	0.4826	ns	Exact
-0.3251	-0.7657 to 0.3233	0.2997	ns	Exact
0.3861	-0.2594 to 0.7931	0.2127	ns	Exact
-0.04039	-0.6126 to 0.5596	0.8939	ns	Exact
-0.02423	-0.6024 to 0.5706	0.9545	ns	Exact
-0.1461	-0.6750 to 0.4819	0.6487	ns	Exact
-0.2067	-0.7076 to 0.4325	0.5168	ns	Exact
-0.3126	-0.7600 to 0.3356	0.3187	ns	Exact
-0.05923	-0.6243 to 0.5465	0.8694	ns	Exact
-0.2403	-0.7248 to 0.4033	0.448	ns	Exact
-0.1418	-0.6726 to 0.4852	0.6593	ns	Exact
-0.2299	-0.7196 to 0.4124	0.4659	ns	Exact
-0.1646	-0.6852 to 0.4672	0.6074	ns	Exact
-0.2627	-0.7360 to 0.3831	0.4067	ns	Exact
0.003503	-0.5844 to 0.5890	0.9961	ns	Exact
-0.06655	-0.6287 to 0.5413	0.8389	ns	Exact
-0.1529	-0.6787 to 0.4766	0.6313	ns	Exact
0.06407	-0.5431 to 0.6272	0.8418	ns	Exact

-0.1211	-0.6608 to 0.5012	0.7049	ns	Exact
-0.1117	-0.6555 to 0.5083	0.7545	ns	Exact
0.1228	-0.4999 to 0.6618	0.7016	ns	Exact
0.4812	-0.1470 to 0.8328	0.1667	ns	Exact
0.01311	-0.5780 to 0.5952	0.9692	ns	Exact
0.3428	-0.3054 to 0.7739	0.273	ns	Exact
0.3316	-0.3168 to 0.7687	0.2898	ns	Exact
0.2491	-0.3954 to 0.7292	0.4286	ns	Exact
-0.08005	-0.6369 to 0.5316	0.8035	ns	Exact
0.02802	-0.5680 to 0.6048	0.9337	ns	Exact
0.1716	-0.4616 to 0.6890	0.592	ns	Exact
0.2172	-0.4235 to 0.7130	0.4946	ns	Exact
0.5469	-0.05859 to 0.8582	0.0686	ns	Exact
0.3341	-0.3142 to 0.7699	0.2854	ns	Exact
0.5371	-0.07242 to 0.8545	0.0746	ns	Exact
0.1403	-0.4864 to 0.6717	0.6591	ns	Exact
0.1611	-0.4700 to 0.6832	0.6146	ns	Exact
0	-0.5867 to 0.5867	>0.9999	ns	Exact
0.5459	-0.06008 to 0.8578	0.069	ns	Exact
0.6162	0.04614 to 0.8835	0.0366	*	Exact
0.6414	0.08768 to 0.8923	0.0282	*	Exact

Significant? (alpha = 0.05)	Number of XY Pairs
No	12
No	12
No	12
No	12
No	12
No	12
No	12
No	12
No	12
Yes	12
No	12
No	12
No	12
No	12
No	12
No	12
No	12
No	12
No	12
No	12
Yes	12
No	12
No	12
No	12
No	12
No	12
No	12
No	12
No	12
No	12
No	12
No	12

No	12
No	12
No	12
No	12
Yes	12
No	12
No	12
No	12
No	12
No	12
No	12
No	12
No	12
No	12
Yes	12
No	12
No	12
No	12
No	12
No	12
No	12
No	12
No	12
No	12
No	12
No	12
No	12
No	12
No	12
Yes	12
No	12

No	12
No	12
No	12
No	12
No	12
Yes	12
No	12
No	12
No	12
No	12
Yes	12
No	12
No	12
No	12
No	12
No	12
Yes	12
No	12
No	12
No	12
No	12
No	12
No	12
No	12
No	12
No	12
No	12
No	12
No	12
No	12
No	12

No	12
No	12
No	12
No	12
Yes	12
No	12
	12
No	12
No	12
No	12
No	12
No	12
No	12
No	12
No	12
No	12
No	12
No	12
No	12
No	12
No	12
No	12
No	12
No	12
No	12
No	12
No	12
No	12
No	12
Yes	12
No	12

No	12
No	12
No	12
No	12
No	12
No	12
No	12
Yes	12
No	12
No	12
No	12
No	12
No	12
No	12
No	12
Yes	12
No	12
No	12
No	12
No	12
No	12
No	12
No	12
No	12
No	12
No	12
No	12
No	12
No	12
No	12
No	12

No	12
No	12
No	12
No	12
No	12
No	12
No	12
No	12
Yes	12
Yes	12
No	12
No	12
No	12
Yes	12
No	12
No	12
No	12
Yes	12
No	12
No	12
No	12
No	12
No	12
No	12
Yes	12
No	12
No	12
No	12
No	12
No	12
No	12

No	12
No	12
No	12
No	12
No	12
No	12
No	12
No	12
No	12
No	12
No	12
No	12
No	12
No	12
No	12
No	12
Yes	12
No	12
No	12
No	12
No	12
No	12
No	12
No	12
No	12
No	12
No	12
No	12
No	12
No	12
No	12

No	12
No	12
No	12
No	12
No	12
No	12
No	12
No	12
No	12
No	12
No	12
No	12
No	12
No	12
No	12
No	12
No	12
No	12
No	12
No	12
No	12
No	12
No	12
No	12
No	12
No	12
No	12
No	12
No	12
No	12
No	12

No	12
No	12
Yes	12
No	12
No	12
No	12
No	12
No	12
No	12
No	12
No	12
No	12
	12
No	12
No	12
No	12
No	12
No	12
No	12
No	12
No	12
No	12
No	12
No	12
No	12
No	12
No	12
No	12
No	12
No	12
No	12

No	12
No	12
No	12
No	12
Yes	12
No	12
No	12
	12
Yes	12
No	12
No	12
No	12
No	12
No	12
No	12
No	12
No	12
No	12
No	12
No	12
No	12
No	12
No	12
No	12
No	12
No	12
No	12
No	12
No	12
No	12
No	12

No	12
No	12
No	12
No	12
No	12
No	12
No	12
No	12
No	12
No	12
No	12
No	12
No	12
No	12
No	12
No	12
No	12
No	12
No	12
Yes	12
Yes	12
